# Genome-wide chromatin accessibility is restricted by ANP32E

**DOI:** 10.1038/s41467-020-18821-x

**Published:** 2020-10-08

**Authors:** Kristin E. Murphy, Fanju W. Meng, Claire E. Makowski, Patrick J. Murphy

**Affiliations:** Department of Biomedical Genetics, Wilmot Cancer Institute, University of Rochester Medical Center, Rochester, NY USA

**Keywords:** Epigenetics, Epigenomics, Gene expression, Histone variants

## Abstract

Genome-wide chromatin state underlies gene expression potential and cellular function. Epigenetic features and nucleosome positioning contribute to the accessibility of DNA, but widespread regulators of chromatin state are largely unknown. Our study investigates how coordination of ANP32E and H2A.Z contributes to genome-wide chromatin state in mouse fibroblasts. We define H2A.Z as a universal chromatin accessibility factor, and demonstrate that ANP32E antagonizes H2A.Z accumulation to restrict chromatin accessibility genome-wide. In the absence of ANP32E, H2A.Z accumulates at promoters in a hierarchical manner. H2A.Z initially localizes downstream of the transcription start site, and if H2A.Z is already present downstream, additional H2A.Z accumulates upstream. This hierarchical H2A.Z accumulation coincides with improved nucleosome positioning, heightened transcription factor binding, and increased expression of neighboring genes. Thus, ANP32E dramatically influences genome-wide chromatin accessibility through subtle refinement of H2A.Z patterns, providing a means to reprogram chromatin state and to hone gene expression levels.

## Abstract (web summary)

Chromatin state underlies cellular function, and transcription factor binding patterns along with epigenetic marks define chromatin state. Here the authors show that the histone chaperone ANP32E functions through regulation of H2A.Z to restrict genome-wide chromatin accessibility and to inhibit gene transcriptional activation.

## Introduction

Access to specific regions of DNA by transcription factors (TFs) and polymerase machinery is critical for the regulation of gene expression. The packaging of DNA, including nucleosome occupancy, positioning, wrapping, and the presence of chromatin modifications, termed “epigenetic marks”, has been implicated in regulating DNA accessibility^1^. However, the degree to which these epigenetic marks regulate TF binding, how, mechanistically, this regulation occurs, and how widespread chromatin state changes impact genome-wide expression patterns are less well established. The histone variant H2A.Z is thought to promote binding of specific TFs, as well as transcriptional activators and repressors^2–5^, but the precise molecular mechanisms by which H2A.Z functions, and whether H2A.Z localization patterns influence genome-wide TF-binding events remains unknown.

The eukaryotic genome is almost completely wrapped by nucleosomes or bound by TFs^6,7^, with less than 5% of total genomic DNA sequence available in a non-nucleosomal context, regardless of cell type^6^. The vast majority of TFs can bind only at these accessible regions. Chromatin state varies considerably between cell types, but how chromatin state transitions occur and the role of epigenetic factors in driving these changes remains unclear^7^. The prevailing model for transitioning between chromatin states relies on passive competition between TFs and histones, but aside from pioneering factors, which bind at limited genomic locations^8,9^, the mechanisms by which the majority of genomic locations are converted from nucleosome bound to TF bound have not been identified.

Previous studies provide examples where H2A.Z localization can promote TF binding, including impacts on OCT4^4^ and FOXA2^5^ in stem cells. H2A.Z presence has also been correlated with DNaseI sensitivity at estrogen-responsive enhancers in human breast cancer cells^2^. Whether H2A.Z nucleosomes are broadly favored for genome-wide TF binding, and the mechanisms by which these nucleosomes might harbor preferred TF-binding sites are presently undetermined. Studies have shown that H2A.Z nucleosomes are more labile than canonical nucleosomes^10,11^. This concept gives rise to several possible ways in which DNA sequences at H2A.Z sites might be more exposed than the DNA of canonical nucleosomes. These include differences in the wrapping of DNA, differences in nucleosome turnover rates, and differences in chromatin stability at H2A.Z nucleosomes^12,13^. Additionally, increased nucleosome remodeling at H2A.Z sites, which has been observed in yeast and plants^5,14–16^, might allow for better access to DNA at H2A.Z nucleosomes compared with canonical nucleosome sites. Notably, it has not yet been established whether these characteristics of H2A.Z are important for mammalian TF binding.

H2A.Z has been most often studied in the context of transcriptional regulation. Studies have shown that decreased polymerase stalling correlates with the presence of H2A.Z at the “+1 nucleosome” region of promoters^17^. In this manner, H2A.Z may promote gene activation by enabling RNA polymerase to overcome chromatin barriers at the earliest stages of gene activation^13,17–21^. However, separate studies have independently demonstrated that H2A.Z can be associated with gene silencing. Loss of H2A.Z causes particular genes to become active, and H2A.Z presence is necessary for establishment of the histone silencing mark H3K27me3 in stem cells^3,4,19,22^. Notably, much of the data supporting the dual role of H2A.Z in transcriptional regulation were acquired in stem cells, in the context of bivalent chromatin^23^, and it is presently unknown whether H2A.Z functions in direct silencing of transcription outside of this context. One additional possibility is that H2A.Z functions differently at distinct genomic locations. Genome-wide H2A.Z patterning is controlled by the H2A.Z incorporation complexes SRCAP and P400, and by the H2A.Z removal complex INO80 (refs. ^24–29^). Additionally, ANP32E has been identified as a chaperone protein that binds the C-terminus of H2A.Z in order to prevent H2A.Z accumulation^30^. Whereas deletion of H2A.Z is lethal, loss of ANP32E is compatible with viability in mice and zebrafish^31,32^. We and others previously found that ANP32E depletion leads to genomic H2A.Z reorganization, including regional H2A.Z accumulation^30,31,33^. Therefore, manipulation of *Anp32e* provides a means to assess locus-specific function of H2A.Z.

Here, we use mouse fibroblasts lacking ANP32E to investigate how genomic H2A.Z organization regulates chromatin state, accessibility to DNA, and gene transcription. We find that modest changes in H2A.Z localization correspond with dramatic and widespread chromatin accessibility changes. Loss of ANP32E results in increased chromatin accessibility at sites where H2A.Z accumulates, but also at sites where high levels of H2A.Z in wild type (WT) remain stable in mutant cells. At thousands of H2A.Z-marked promoters, we find that H2A.Z expands when ANP32E is absent, spreading from the +1 nucleosome to the −1 nucleosome, and this expansion is associated with a dramatic increase in chromatin accessibility. In this regard, we determine that H2A.Z localization conforms to a hierarchy surrounding the transcription start site (TSS), and this hierarchy distinguishes strength of adjacent nucleosome positioning as well as TF motif accessibility. Combined depletion of H2A.Z and ANP32E largely reverses these outcomes, further indicating that ANP32E regulates chromatin state through antagonism of H2A.Z. Finally, we find that ANP32E is necessary for activation of genes involved in cellular proliferation, and for silencing of genes involved in developmental differentiation. Thus, we establish that ANP32E controls genome-wide H2A.Z enrichment levels, and positioning of H2A.Z around the TSS, in order to regulate promoter accessibility and hone widespread gene expression levels.

## Results

### H2A.Z resides at active genes and highly accessible chromatin regions

**Fig. 1:**
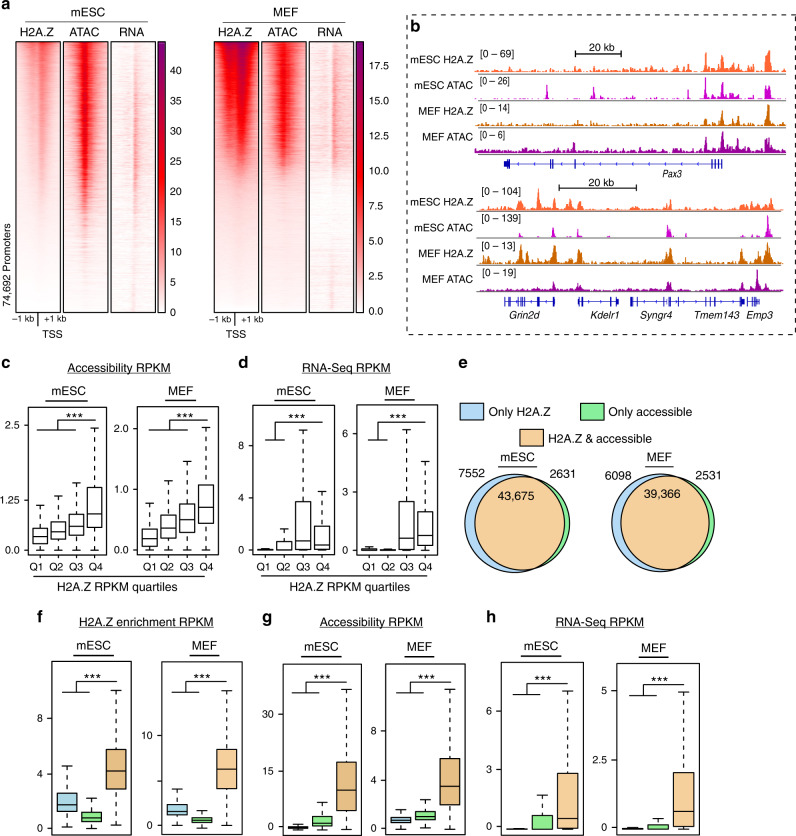
Genome-wide H2A.Z correlation with chromatin accessibility and gene expression across multiple cell types. **a** Heatmaps of normalized H2A.Z enrichment (ChIP-Seq), chromatin accessibility (ATAC-Seq), and RNA expression signals (RNA-Seq) at promoter regions (TSSs ± 1 kb). Rows in each heatmap are ordered by decreasing H2A.Z signal. **b** A gallery of genome browser snapshots for representative genomic loci depicting the correlation of H2A.Z and chromatin accessibility (ATAC). Normalized read counts are shown. **c** Boxplots of chromatin accessibility RPKM values for H2A.Z quartiles. A random sampling of regions (300 bp, *n* = 50,000) of mouse genome are used. **d** Boxplots of RNA expression RPKM values for H2A.Z quartiles at promoter regions (TSS ± 1 kb, *n* = 74,692 promoters). **e** Venn diagram showing overlap of H2A.Z peaks and chromatin accessibility peaks at promoter regions (TSS ±1 kb) for each cell type. The number of peaks is labeled for each category (only H2A.Z occupied, light blue; only accessible promoter regions, green; and both H2A.Z occupied and accessible regions, orange). **f**–**h** Boxplots of RPKM values of H2A.Z enrichment (**f**), chromatin accessibility (**g**), and RNA expression (**h**) for each category. Colors and number of regions in each category are indicated in panel **e**. Boxes = interquartile ranges, middles = medians, whiskers = 1.5× the interquartile range, adjusted *p* values from pairwise two-sided Wilcoxon rank-sum test, ***p < 0.0001.

To begin investigating our hypothesis that H2A.Z contributes to gene activation and chromatin accessibility, we assessed two embryonic mouse cell types, mouse embryonic stem cells (mESCs) and mouse embryonic fibroblasts (MEFs). We found a high degree of overlap between H2A.Z and high chromatin accessibility at promoter regions (defined as regions surrounding the TSS +/− 1 kb) (Fig. 1a) as well as H2A.Z sites genome wide (Fig. 1b and Supplementary Fig. 1a) (sources of publicly available datasets in Supplementary Table 1). After partitioning the entire genome based on H2A.Z enrichment, we found that the highest levels of H2A.Z coincided with the highest degree of chromatin accessibility (Fig. 1c), and the most accessible regions had the highest levels of H2A.Z (Supplementary Fig. 1b). This trend was even more apparent when we limited our analysis to promoters. Here, H2A.Z-marked promoters were more highly expressed (Fig. 1d), and the most accessible promoters were almost always marked by H2A.Z (Fig. 1e). Enrichment levels were significantly lower at locations where H2A.Z and chromatin accessibility occurred alone compared with where they occurred together (Fig. 1f, g). Additionally, gene expression was considerably higher for accessible H2A.Z-marked promoters compared with promoters lacking either of these attributes (Fig. 1h).

As noted, prior studies demonstrated that H2A.Z is necessary for recruitment of epigenetic silencing factors, including the PRC2 complex^3,4,22^, but in our initial analyses, we found that H2A.Z was strongly correlated with gene activation. Thus, we sought to further investigate how H2A.Z and chromatin accessibility associate with the activating epigenetic marks H3K4me3 and H3K27ac, and with the silencing mark H3K27me3. Here we found that H2A.Z is associated with bivalent chromatin marks H3K27me3 and H3K4me3 in mESCs (Supplementary Fig 1c, top), consistent with prior studies^3,4,18,19^, but in MEFs, H2A.Z and H3K27me3 occur mostly at separate genomic locations (Supplementary Fig 1d). In line with the observed cell-type-specific overlap of H2A.Z and H3K27me3, we found that H2A.Z was more highly correlated with gene activation in MEFs than in mESCs (Fig. 1c, bottom). We also found cell-type-specific enhancer localization differences comparing MEFs with mESC (enhancers were defined as all H3K27ac-occupied regions more than 2 kb away from promoters), but unlike our measurements of H3K27me3, there was considerable overlap of H3K27ac, H2A.Z, and chromatin accessibility, regardless of cell type (Supplementary Fig 1e, f). These results indicate that H2A.Z is consistently correlated with chromatin accessibility, as well as with marks of gene activation H3K4me3 and H3K27ac, but H2A.Z is poorly associated with the gene silencing mark H3K27me3 in differentiated MEFs.

### Loss of ANP32E leads to a widespread increase in chromatin accessibility

**Fig. 2:**
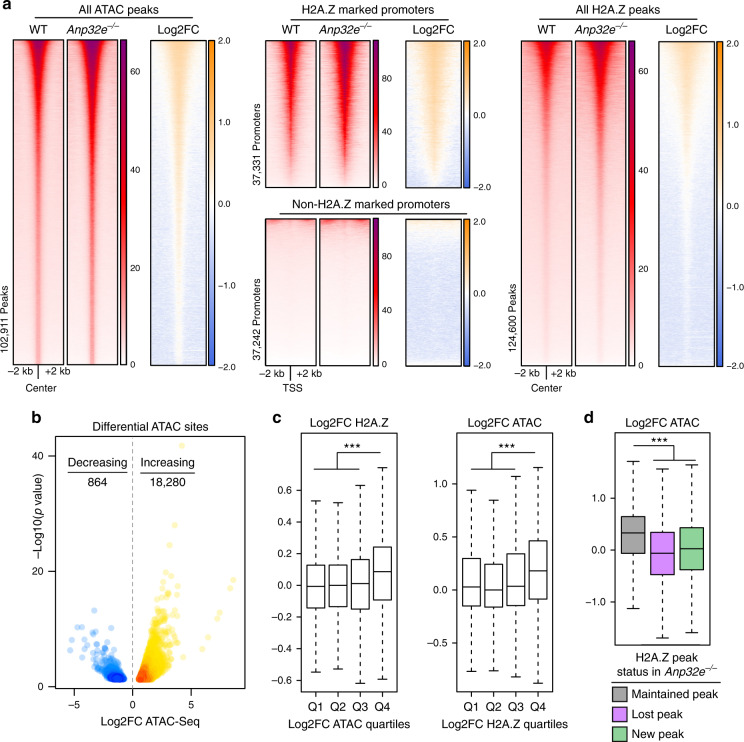
Genome-wide chromatin accessibility increases upon ANP32E loss. **a** Heatmaps of normalized chromatin accessibility reads (ATAC) and log2FC of ATAC-Seq demonstrating a genome-wide increase in chromatin accessibility at all accessible sites (left panel), H2A.Z-marked promoter regions (TSS ± 1 kb, middle panel), and H2A.Z peaks (right panel) in *Anp32e*^−/−^ MEFs compared to WT MEFs. Union peaks of WT and *Anp32e*^−/−^ MEFs chromatin accessibility and H2A.Z enrichment peaks were used for plotting, and heatmaps are ordered by total ATAC signal. **b** Volcano plot of differential ATAC sites in *Anp32e*^−/−^ MEFs. Differential ATAC sites were identified by DiffBind using ATAC union peaks and triplicates of ATAC-Seq with default settings. Increased ATAC sites are shown in orange (log2FC > 0.5 and adjusted *p* < 0.05, *n* = 18,280), and decreased ATAC sites are shown in blue (log2FC < −0.5 and adjusted *p* < 0.05, *n* = 864). Color intensity represents density of points in the volcano plot (*p* values from Wald test and adjusted for multiple testing). **c** Boxplots of log2FC of H2A.Z enrichment of ATAC quartiles (left) and chromatin accessibility of H2A.Z quartiles (right) at promoter regions (TSS ± 1 kb, *n* = 74,692) in *Anp32e*^−/−^ compared to WT MEFs. **d** Boxplots of log2FC of chromatin accessibility at shared H2A.Z peaks (*n* = 57,975), WT unique H2A.Z peaks (*n* = 28,740), and *Anp32e*^−/−^ unique H2A.Z peaks (*n* = 37,961). Adjusted *p* values from a two-sided Student’s *t*-test, boxes = interquartile ranges, middles = medians, whiskers = 1.5× the interquartile range, ***p < 0.0001.

Having found a strong association of H2A.Z with highly accessible chromatin, we next sought to functionally test whether increasing H2A.Z enrichment levels, and altering H2A.Z genomic locations, would cause corresponding changes in chromatin accessibility. In separate mouse, human, and zebrafish studies, loss of the histone chaperone ANP32E resulted in genomic H2A.Z mislocalization^30,31,33^. Therefore, we chose to investigate how chromatin accessibility changed in response to H2A.Z localization changes in primary MEFs lacking ANP32E (Supplementary Fig. 2a)^30,32^. Our initial analyses took place at the genome-wide level, where unbiased clustering revealed strong concordance of biological replicates (Supplementary Fig. 2b). Loss of ANP32E resulted in dramatic and widespread increases in chromatin accessibility across the genome (Fig. 2a, left). In fact, we observed heightened chromatin accessibility at nearly 95% of all differentially accessible sites (Fig. 2b and Supplementary Fig. 2c). We did not expect such sweeping chromatin accessibility increases in *Anp32e* null MEFs based on prior reports that ANP32E loss leads to ectopic H2A.Z incorporation at approximately 4000 new sites^30^. In contrast, we found that accessibility increased significantly at more than 18,000 sites, and we observed modest accessibility increases at almost all of the H2A.Z-marked promoters (Fig. 2a, middle) and H2A.Z peaks (Fig. 2a, right).

If H2A.Z functions to promote chromatin accessibility, then changes in H2A.Z levels might result in differing magnitudes of accessibility changes. We therefore investigated whether the observed chromatin accessibility increases in *Anp32e* null MEFs were accompanied by changes in H2A.Z levels. ANP32E loss led to an overall increase in H2A.Z enrichment, but we also found many sites where H2A.Z was either unchanged or reduced (Supplementary Fig. 2d). Regions with the greatest increase in H2A.Z were indeed those with the greatest increase in chromatin accessibility, and likewise, regions where chromatin became most accessible were also those where H2A.Z increased the most (Fig. 2c). However, the strongest increases occurred at sites where H2A.Z was maintained between WT and mutant cells, as compared with new H2A.Z peaks (Fig. 2d and Supplementary Fig 2e). We also found modest increases in accessibility at a subset of regions where H2A.Z was reduced (Supplementary Fig 2d), but measurements of fold change revealed that accessibility at these regions remained mostly unchanged (Fig. 2d). These results indicate that changes in H2A.Z abundance do indeed confer changes in chromatin accessibility, but additional factors aside from H2A.Z enrichment might also contribute to accessibility changes.

### H2A.Z hierarchy changes correspond with accessibility changes

**Fig. 3:**
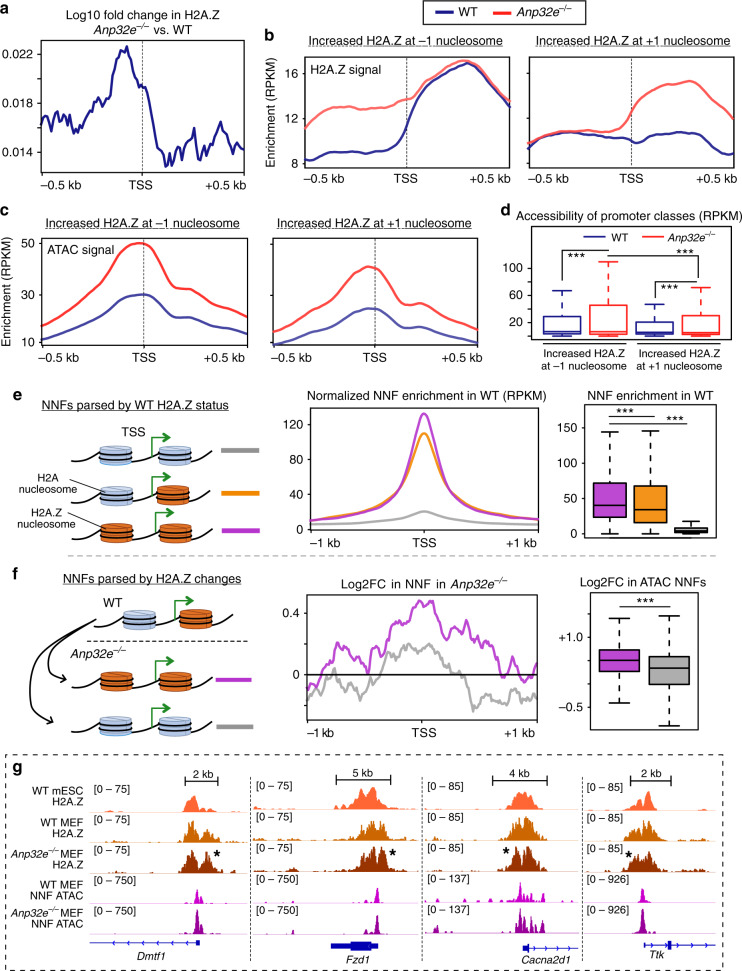
Changes in H2A.Z correspond with a positional hierarchy at promoters. **a** Change in H2A.Z showing an overall increase upstream of the TSS (indicated by a dotted line). *Y*-axis value represents log10FC in H2A.Z levels comparing *Anp32e*^−/−^ to WT MEFs. **b** Aggregate H2A.Z enrichment plots in WT (blue) and *Anp32e*^−/−^ (red) at defined promoter categories. Regions where H2A.Z increased at −1 nucleosome are in the left panel, and regions where H2A.Z increased at +1 nucleosome are in the right panel (see details in “Methods”). **c** Aggregate total ATAC-Seq enrichment plots for WT and *Anp32e*^−/−^ signals at defined categories of promoters: those where H2A.Z increased at −1 nucleosome (left panel) and those where H2A.Z increased at +1 nucleosome (right panel). **d** Boxplot of chromatin accessibility signals of WT MEFs and *Anp32e*^−/−^ MEFs at defined categories of promoters (TSS ± 500 bp, *n* = 74,692 total promoters): those where H2A.Z increased at −1 nucleosome and those where H2A.Z increased at +1 nucleosome. **e** Aggregate profile plot and boxplot of non-nucleosome fragments (NNF) of WT MEF chromatin accessibility reads for gene promoters parsed by H2A.Z status. Higher NNF distribution at “Class Two” promoters (purple, *n* = 10,965) than “Class One” (orange, *n* = 15,401) or “Class Zero” promoters (gray, *n* = 48,199). Cartoon (left) indicates Class transitions, with aggregate plot (middle) and boxplots (right) displayed. **f** Aggregate profile plot and boxplot of log2FC of small fragment chromatin accessibility reads (NNF non-nucleosome fragments) for two clusters of genes: those that gain H2A.Z from “Class One” to “Class Two” promoter (purple, *n* = 2143) and those that lose H2A.Z from “Class One” to “Class Zero” promoter (gray, *n* = 1858) compared to *Anp32e*^−/−^ MEFs to WT MEFs. Log2FC of small fragment chromatin accessibility reads (<150 bp) was generated by comparing *Anp32e* null chromatin accessibility NNF reads to WT chromatin accessibility NNF reads. Cartoon (left) indicates Class transitions, with aggregate plot (middle) and boxplots (right) displayed. **d**–**f** For boxplots, boxes = interquartile ranges, middles = medians, whiskers = 1.5× the interquartile range, adjusted *p* values from pairwise two-sided Wilcoxon rank-sum test, and ***p < 0.0001. **g** Genome browser snapshot of H2A.Z enrichment and non-nucleosome fragments (NNF) of ATAC-Seq in WT mESCs, WT MEFs, and *Anp32e*^−/−^ MEFs. Star denotes examples for increased H2A.Z upstream of the TSS.

We next sought to identify additional H2A.Z-specific chromatin changes that might account for the dramatic chromatin accessibility changes we observed. Several studies have reported H2A.Z enrichment at promoters flanking the TSS, including the first nucleosome located upstream of the TSS, termed the “−1 nucleosome” position, and the first nucleosome located downstream of the TSS, termed the “+1 nucleosome” position^19,34–36^. The 200–500 bp regions located between these two nucleosome positions is often referred to as the nucleosome-depleted region (NDR). In yeast, the H2A.Z installation enzyme Swr1 has been reported to localize at NDR sites to install H2A.Z at the +1 nucleosome of promoters^36–38^. These observations prompted us to investigate whether H2A.Z localization on either side of the TSS was impacted by ANP32E loss in MEFs. Indeed, in the absence of ANP32E, H2A.Z accumulated mostly upstream of the TSS, near the −1 nucleosome position (Fig. 3a). In order to better understand how H2A.Z might accumulate specifically at these sites, we partitioned promoters based on WT H2A.Z levels. Here we found there to be minimal H2A.Z changes in *Anp32e*^−/−^ cells for promoters with the highest levels of H2A.Z in WT (Supplementary Fig. 3a). Alternatively, for promoters with low H2A.Z levels in WT, H2A.Z tended to accumulate broadly surrounding the TSS. For promoters with moderate H2A.Z levels in WT, H2A.Z accumulated mostly at the −1 nucleosome, suggesting that ANP32E might regulate positioning of H2A.Z on either side of the TSS. To investigate this result further, we categorized H2A.Z-marked promoters based on relative changes in H2A.Z enrichment. Remarkably, at promoters where H2A.Z increased specifically at the −1 position, high levels of H2A.Z were maintained at the +1 nucleosome position in both mutant and WT conditions (Fig. 3b, left and Supplementary Fig. 3b). Alternatively, when H2A.Z accumulated specifically at the +1 nucleosome, these promoters generally lacked H2A.Z in WT MEFs (Fig. 3b, right and Supplementary Fig. 3b). Taken together, these results indicate that H2A.Z localization around the TSS adheres to a positional hierarchy, where de novo H2A.Z accumulates preferentially at the +1 nucleosome, and if H2A.Z is already present at the +1 nucleosome, additional H2A.Z accumulates at the −1 nucleosome.

We next asked whether ANP32E regulates H2A.Z localization at specific classes of gene promoters according to this positional hierarchy. To address this, we classified promoters based on H2A.Z localization changes in *Anp32e*^−*/*−^ MEFs relative to WT, and then assessed whether functionally distinct gene sets independently segregated (Supplementary Fig. 3c). Promoters lacking H2A.Z were defined as “Class Zero”, promoters with H2A.Z at only the +1 position were defined as “Class One,” and promoters with H2A.Z at both TSS flanking positions were defined as “Class Two”. Indeed, gene ontology (GO) analysis revealed that expansion of H2A.Z to the −1 nucleosome (going from Class One to Class Two) in *Anp32e* null MEFs occurred at genes associated with developmental differentiation and mRNA translation (Supplementary Fig. 3d), while reduction in H2A.Z was associated with proliferation, locomotion, and migration (Supplementary Fig. 3e). These results support a role for ANP32E in restricting H2A.Z expansion at promoters of genes involved in distinct biological processes, prompting us to speculate that similar transitions between H2A.Z classes might occur during normal cellular differentiation. As a proxy for differentiation, we compared WT mESCs with WT MEFs, and again parsed promoters into defined H2A.Z-positioning classes. Here we observed a preference for H2A.Z expansion in MEFs as compared with mESCs (Supplementary Fig. 3c, bottom right). Similar to the ANP32E-dependent H2A.Z changes we observed, distinct GO terms related to mRNA translation and cell signaling (among other terms) were identified for genes where promoter H2A.Z localization changed (Supplementary Fig. 3f). Consistent with the possibility that ANP32E levels and H2A.Z positioning are important for defining cellular function during differentiation, comparisons of RNA-Seq datasets indicate that *Anp32e* mRNA is considerably lower in MEFs compared with mESCs (Supplementary Fig. 3g).

Having found that ANP32E loss impacts H2A.Z localization patterns at gene promoters, we next asked whether these changes confer chromatin state changes surrounding the TSS. We again categorized promoters based on H2A.Z changes, and found that loss of ANP32E led to an increase in accessibility over the NDR when H2A.Z increased at either the −1 or +1 position (Fig. 3c, d). We next wondered whether differences in H2A.Z promoter classes associated with differences in chromatin accessibility. For better resolution over the NDR, we focused on non-nucleosome chromatin fragments (NNFs), which are typically enriched at the TSS^39^. These NNFs can be quantified from paired-end ATAC-Seq data^39^ by selecting fragments smaller than 150 bp. Indeed, in WT MEFs, Class Two promoters had the highest NNF levels, and Class Zero had the lowest NNF levels (Fig. 3e). Additionally, transitioning from Class One to Class Two in ANP32E null MEFs resulted in a significant increase in NNFs at promoters (Fig. 3f, example in 3g). Taken together these data suggest that levels of chromatin accessibility are altered by ANP32E through regulation of H2A.Z positioning at promoter regions.

### Reduction in H2A.Z partially rescues impacts of ANP32E loss on chromatin accessibility

**Fig. 4:**
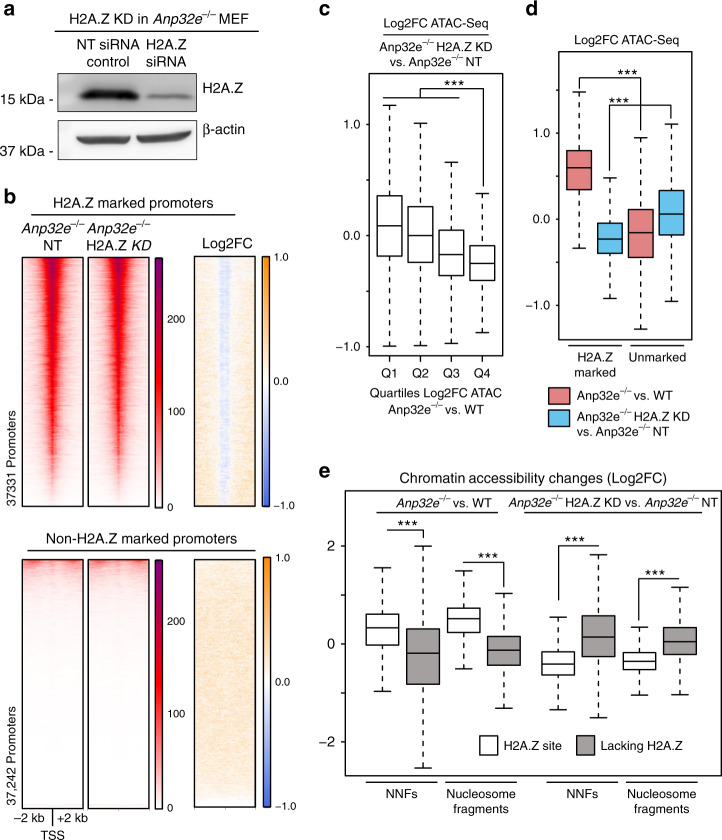
Chromatin accessibility changes upon Anp32e loss depend on H2A.Z. **a** Representative western blot of H2A.Z indicates knock down of H2A.Z after siRNA transfection compared with non-targeting (NT) siRNA. β-Actin is used as the loading control (*n* = 4 replicates). **b** Heatmaps of normalized chromatin accessibility reads for control siRNA and H2A.Z siRNA treated *Anp32e*^−/−^ MEFs and log2FC (H2A.Z siRNA/control siRNA) at H2A.Z-marked promoter regions (top panel) and non-H2A.Z-marked promoter regions (bottom panel). Heatmaps of ATAC-Seq are ordered by total ATAC signal, and the same ranked regions were used to generate log2FC heatmaps. **c** Boxplot of log2FC ATAC-Seq (H2A.Z knockdown *Anp32e*^−/−^ ATAC-Seq versus non-targeting control *Anp32e*^−/−^ ATAC-Seq) at quartiles of promoter regions (TSS ± 1 kb, *n* = 74,692). Quartiles of promoters were generated based on log2FC of *Anp32e*^−/−^ MEF ATAC-Seq to WT MEF ATAC-Seq, where Q4 have the highest increase of ATAC-Seq. **d** Boxplot of log2FC ATAC-Seq (*Anp32e*^−/−^ MEF ATAC-Seq versus WT MEF ATAC-Seq, shown in red) and (H2A.Z knockdown *Anp32e*^−/−^ ATAC-Seq versus non-targeting control *Anp32e*^−/−^ ATAC-Seq, shown in blue) at H2A.Z-marked promoter regions (TSS ± 1 kb, *n* = 74,692) and non-H2A.Z-marked promoter regions (TSS ± 1 kb). **e** Boxplot of log2FC NNF (non-nucleosome fragments, <150 bp) and nucleosome fragments (>150 bp) of ATAC-Seq at promoter regions (TSS ± 1 kb, *n* = 74,692) for H2A.Z-marked sites (shown in unfilled boxes) and non-H2A.Z-marked sites (shown in filled boxes). **c**–**e** For boxplots, boxes = interquartile ranges = medians, whiskers = 1.5× the interquartile range, adjusted *p* values from pairwise two-sided Wilcoxon rank-sum test, and ***p < 0.0001.

Because we found that chromatin accessibility changes in ANP32E-deficient cells corresponded with changes in H2A.Z levels and H2A.Z positioning surrounding the TSS, we next sought to investigate whether these accessibility changes were dependent on H2A.Z. Thus, we knocked down H2A.Z in ANP32E-deficient cells (Fig. 4a), and repeated our measurements of chromatin accessibility. Indeed, H2A.Z depletion caused chromatin accessibility to decrease at the NDR of H2A.Z-marked promoters (Fig. 4b). Consistent with a role for H2A.Z in regulation of accessibility, the genomic regions where accessibility increased the most in ANP32E-deficient cells, had the greatest reduction in accessibility upon H2A.Z depletion (Fig. 4c). This overall reversal in accessibility occurred at sites marked by H2A.Z, but not at unmarked sites (Fig. 4b, d). We then measured changes on a locus-by-locus manner and found that accessibility was mostly reversed if H2A.Z was present (85% of sites) at promoters which became more accessible in *Anp32e*^−*/*−^ MEF (Supplementary Fig. 4a, b). This trend was also apparent outside of promoters, but to a lesser extent (67% of sites) (Supplementary Fig. 4c, d). Depletion of H2A.Z caused reciprocal changes in chromatin accessibility regardless of whether we measured NNFs or nucleosome sized fragments (Fig. 4e). Taken together, these data indicate that ANP32E mediated regulation of chromatin accessibility occurs at H2A.Z sites, in an H2A.Z-dependent manner, even though loss of ANP32E caused only modest changes in H2A.Z levels.

**Fig. 5:**
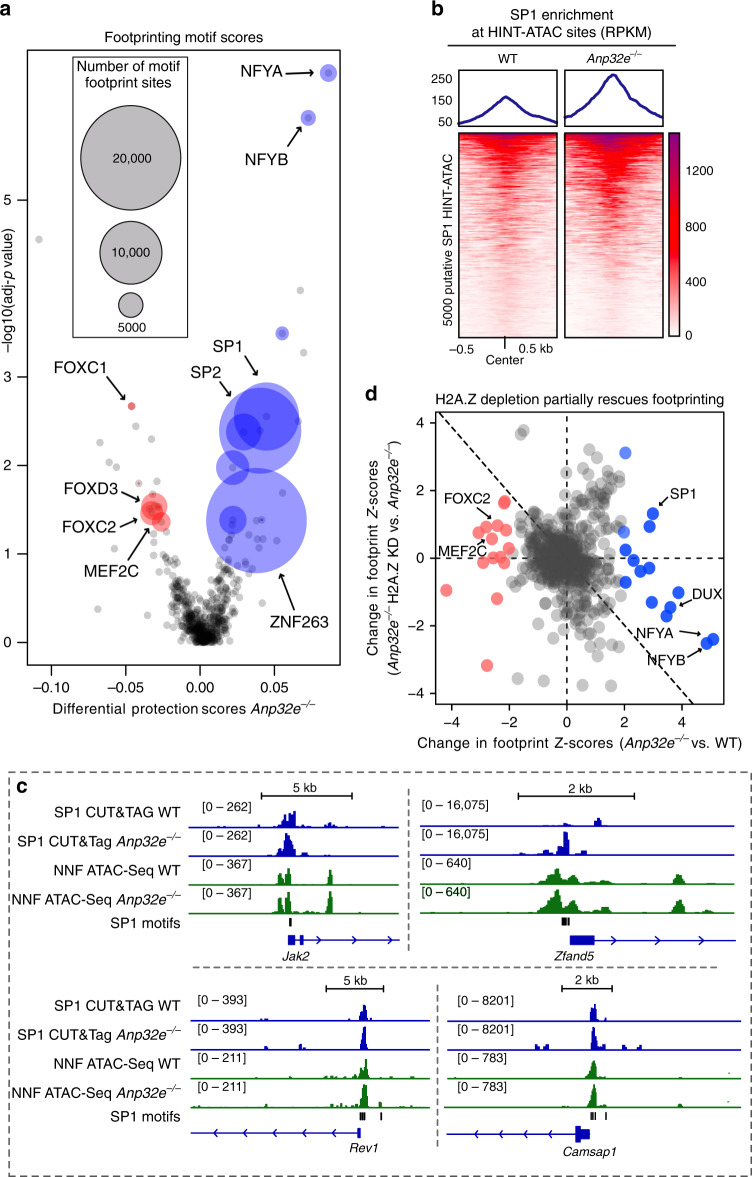
Hint-ATAC identifies differential transcription factor footprints in *Anp32e*^−/−^ MEFs. **a** Differential transcription factor footprints comparing *Anp32e*^−/−^ MEFs to WT MEFs chromatin accessibility reads. Footprints of transcription factors are identified by HINT-ATAC using chromatin accessibility reads and narrowPeak files (see “Methods”). Each point represents an individual TF and the area of the point represents number of footprints identified for the corresponding TF. TFs with increased protection score in *Anp32e*^−/−^ are shown in blue and TFs with decreased protection score in *Anp32e*^−/−^ are shown in red. Names of selected TFs with significant differential activity values are labeled. **b** Heatmap of SP1 CUT&Tag RPKM values showing increased SP1 signal at putative SP1 HINT-ATAC sites in *Anp32e*^−/−^ MEFs. **c** Genome browser views of SP1 CUT&Tag profiles and non-nucleosome fragments (NNF) of ATAC-Seq at representative loci. **d** Scatterplot of HINT-ATAC transcription factor footprints *Z*-scores. Each point represents a transcription factor motif identified by HINT-ATAC, and axis represents change in footprints *Z*-score, where positive *Z*-score indicates increased footprints and negative *Z*-score indicates decreased footprints. Significantly increased TF footprints in *Anp32e*^−/−^ MEFs versus WT MEFs are labeled in blue, while significantly decreased TF footprints are labeled in red.

Our findings that loss of ANP32E led to an increase in chromatin accessibility for NNFs at NDRs prompted us to investigate whether TFs might bind more effectively in response to ANP32E loss. To identify putative TF-binding events, we performed footprinting analysis using HINT-ATAC-Seq^40^. Our assessment revealed footprinting over several known TF motifs, which are differentially protected in the *Anp32e*^−/−^ cells (Fig. 5a). HINT-ATAC protection scores correspond with difference in cleavage events between the footprint and flanking regions, and are commonly used for assessing footprinting^40,41^. We found there to be far more footprint sites genome-wide identified for TF motifs with positive HINT scores, than those with negative scores (Fig. 5a), indicative of an overall increase in TF binding. Examples of motifs identified include those for NF-YB and SP1, which are known to physically interact and have roles in developmental differentiation^42–47^. We validated our HINT-ATAC approach using Cleavage Under Targets and Tagmentation^48^ (CUT&Tag) to measure SP1 chromatin binding in *Anp32e*^−/−^ cells (Fig. 5b). Loss of ANP32E led to an overall increase in SP1 binding both at accessible SP1 motifs (Supplementary Fig. 5a), and at footprints identified by HINT-ATAC (Fig. 5b and Supplementary Fig. 5b). Having found that increases in NNFs were partially dependent on H2A.Z, we next repeated our HINT-ATAC measurements in cells lacking ANP32E and depleted for H2A.Z. Consistent with a role for H2A.Z in TF binding, footprint scores for many of the HINT-ATAC sites identified in *Anp32e*^−*/*−^ cells changed in an opposite manner upon H2A.Z loss, including two-thirds of statistically significant sites (Fig. 5d and Supplementary Fig. 5c). Motifs for SP1 were not among this group, suggesting that additional factors impact SP1 binding. Taken together, these results provide strong evidence that ANP32E loss causes increased TF binding and many of these increases occur in an H2A.Z-dependent manner.

### ANP32E loss leads to increased SMARCA4 enrichment and improved nucleosome positioning

**Fig. 6:**
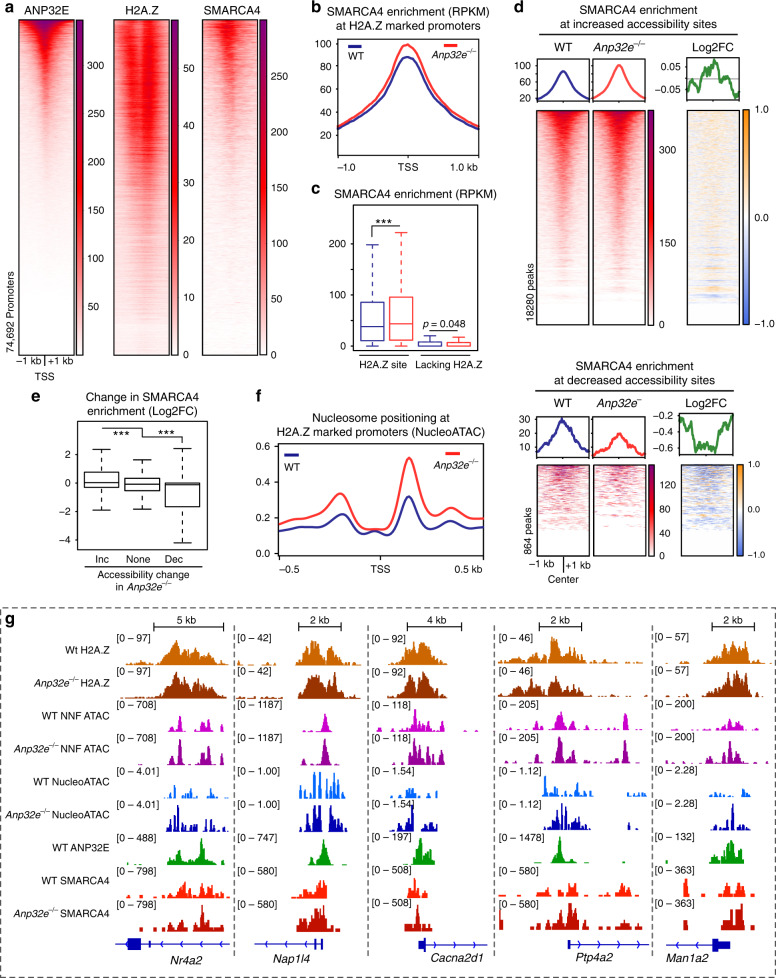
Increased SMARCA4 binding in *A*np32e^−/−^ MEFs. **a** Heatmaps of read count normalized enrichment for ANP32E (CUT&Tag), H2A.Z (ChIP-Seq), and SMARCA4 (CUT&Tag) depicting strong overlaps at promoter regions (TSS ± 1 kb). **b** Aggregate profile plot of SMARCA4 CUT&Tag signal showing increased SMARCA4 enrichment at H2A.Z-marked promoters in *Anp32e*^−/−^ MEFs. **c** Boxplot of SMARCA4 CUT&Tag enrichment signal of WT MEFs and *Anp32e*^−/−^ MEFs at H2A.Z-marked promoters and non-H2A.Z-marked promoters (*n* = 74,692 total promoters, adjusted *p* values from a two-sided Student’s *t*-test, boxes = interquartile ranges, middles  = medians, whiskers = 1.5× the interquartile range, ***p < 0.0001). **d** Heatmaps of SMARCA4 CUT&Tag signal in WT MEFs and *Anp32e*^−/−^ MEFs and log2FC of SMARCA4 showing increased SMARCA4 signal at ATAC sites with increased chromatin accessibility (top panel), and decreased SMARCA4 signal at ATAC sites with decreased chromatin accessibility (bottom panel). **e** Boxplot of log2FC of SMARCA4 enrichment at ATAC sites with increased chromatin accessibility, ATAC sites with no significant change, and ATAC sites with decreased chromatin accessibility. (*n* = 102,911 total ATAC peaks, adjusted *p* values from a two-sided Student’s *t*-test, boxes = interquartile ranges, middles = medians, whiskers = 1.5× the interquartile range, ***p < 0.0001). **f** Aggregate profile plot of nucleosome positioning identified by NucleoATAC at H2A.Z-marked promoters. **g** Genome browser snapshots for representative genomic loci depicting H2A.Z enrichment, chromatin accessibility NNF reads (ATAC), NucleoATAC signal, ANP32E CUT&Tag signal, and SMARCA4 CUT&Tag signal in *Anp32e*^−/−^ MEFs and WT MEFs.

ANP32E may restrict chromatin accessibility through changes in nucleosome turnover, wrapping, or remodeling, among other possibilities. Studies of nucleosome remodelers in yeast indicate that SWI/SNF and ISWI family nucleosome remodelers preferentially reposition H2A.Z-containing nucleosomes^14,15^. If conserved in mammals, this is a potential pathway by which ANP32E might function to control chromatin patterns. To investigate this, we performed CUT&Tag measuring the genomic localization for ANP32E and SMARCA4 (also known as BRG1), the closest mouse homolog to the RSC catalytical subunit^49–51^. Strikingly, SMARCA4 and ANP32E were enriched over the TSS, and both factors occupied promoters where H2A.Z is enriched (Fig. 6a). Pairwise comparisons of these datasets revealed a high degree of overlap between H2A.Z, SMARCA4, and ANP32E at promoters (Supplementary Fig. 6a), and assuredly, SMARCA4 localization was very similar to published SMARCA4 patterns generated through ChIP-Seq^50^ (Supplementary Fig 6b). The correlation between SMARCA4 and H2A.Z was more moderate at enhancers compared with promoters (Supplementary Fig. 6c). Loss of ANP32E caused a modest but statistically significant increase in SMARCA4 enrichment at H2A.Z-marked promoters (Fig. 6b, c). Additionally, SMARCA4 enrichment tended to increase at regions where chromatin accessibility increased, and enrichment decreased where accessibility was reduced (Fig. 6d, e). Based on these SMARCA4 changes, we next reasoned that nucleosome positioning surrounding the TSS might also be impacted by ANP32E loss. Indeed, using NucleoATAC-Seq^52^ we found that nucleosome positioning is strongest at H2A.Z-marked promoters, and positioning improved further at these sites upon ANP32E loss (Fig. 6f and Supplementary Fig. 6d, e). Parsing promoters based on H2A.Z changes further revealed that nucleosome positioning was indeed strongest at promoters where H2A.Z increased in *Anp32e*^−/−^ cells (Supplementary Fig. 6f). Taken together, these results are consistent with a nucleosome remodeling mechanism, where H2A.Z promotes increased nucleosome positioning flanking the TSS, stimulating chromatin accessibility at the NDR (examples in Fig. 6g).

### ANP32E loss leads to dysregulation of genes involved in differentiation

**Fig. 7:**
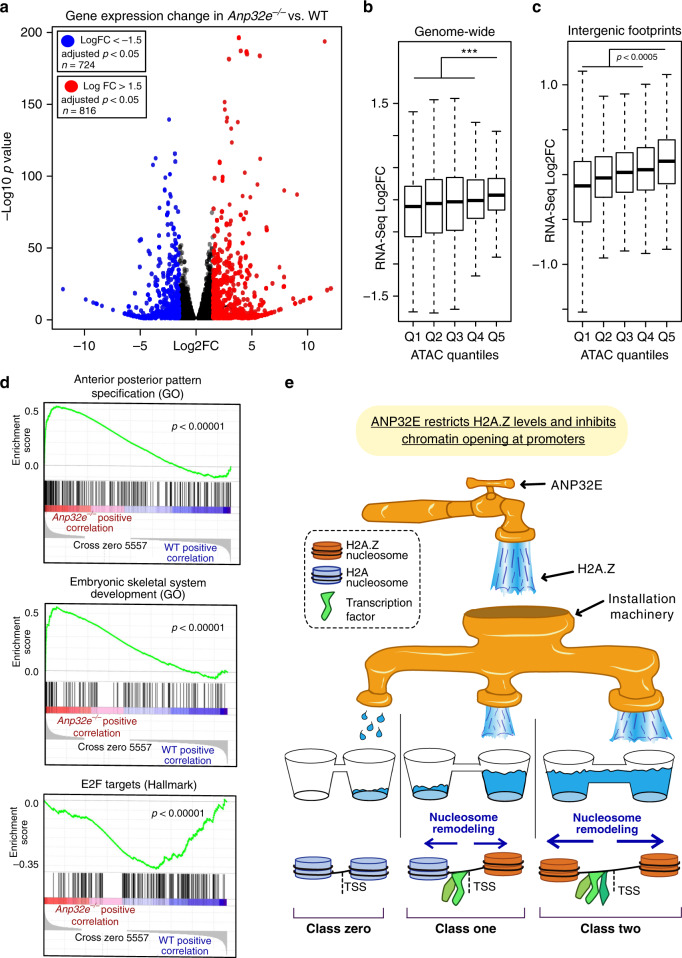
Differential gene expression profiles in *Anp32e*^−/−^ MEFs. **a** Volcano plot of differential gene expression comparing *Anp32e*^−/−^ to WT MEFs. Each point represents the average value of one transcript. Transcripts with significantly increased expression (log2FC > 1.5 and adjusted *p* < 0.05) in *Anp32e*^−/−^ MEFs are shown in red and those with significantly decreased expression (log2FC < −1.5 and adjusted *p* < 0.05) are shown in blue (*p* values from Wald test and adjusted for multiple testing). **b** Boxplots for log2FC of RNA expression at promoter regions (TSS ± 1 kb, *n* = 74,692) compared with *Anp32e*^−/−^ MEFs to WT MEFs. Quantiles are based on fold change in ATAC-Seq enrichment (adjusted *p* values from a two-sided Student’s *t*-test, boxes =  interquartile ranges, middles = medians, whiskers = 1.5× the interquartile range, ***p < 0.0001). **c** Boxplots for log2FC of RNA expression at genes (*n* = 74,692) nearest to intergenic TF footprints comparing *Anp32e*^−/−^ MEFs to WT MEFs. Quantiles are based on fold change in ATAC-Seq enrichment. Statistical significance is assessed with a two-sided Student’s *t*-test. (boxes = interquartile ranges, middles = medians, whiskers = 1.5× the interquartile range, adjusted *p* values from a two-sided Student’s *t*-test). **d** Gene set enrichment analysis of *Anp32e*^−/−^ compared to WT MEF RNA expression data revealed several gene sets. Examples are shown (nominal *p* values were generated based on statistical significance of individual gene sets without correction). **e** A model for ANP32E regulating genomic H2A.Z localization and chromatin accessibility dynamics. ANP32E regulates Hi2A.Z abundance, and installation occurs in a targeted hierarchical manner. At locations where targeted installation is high, H2A.Z is installed at the +1 nucleosome position until it reaches maximum occupancy, and then H2A.Z is installed at the −1 nucleosome position. At locations where targeting is moderate, H2A.Z is installed only at the +1 nucleosome position, and where targeting is low, H2A.Z is absent. Where H2A.Z is most abundant, chromatin remodelers readily position nucleosomes flanking the nucleosome-depleted region, TF binding increases, and neighboring gene expression increases.

We expected that genome-wide transcriptional changes would occur as a consequence of the increased accessibility and TF footprinting that we observed. We therefore assessed genome-wide RNA expression levels (Supplementary Fig. 7a), and identified 1540 differentially expressed genes in the *Anp32e*^−/−^ cells (Fig. 7a). Regions with increased accessibility occurred proximal to up-regulated genes, and regions with reduced accessibility occurred proximal to down-regulated genes (Fig. 7b). This trend was maintained even for distal intergenic motifs identified by footprinting analysis (Fig. 7c), indicating that the ANP32E mediated changes at intergenic regulatory regions, such as enhancers, may contribute to the gene expression changes we observed. Similar to our investigation of promoters undergoing H2A.Z expansion, GO analysis revealed that genes critical for development were highly dysregulated (Supplementary Fig. 7b). Genes involved in pattern formation tended to be up-regulated, while genes involved in cell signaling tended to be down-regulated. Notably, GO terms associated with morphogenesis were present for both up- and down- regulated groups. Gene set enrichment analysis (GSEA) identified several developmentally important gene sets in which expression was significantly increased, and we found down-regulated genes to be associated with cell cycle and proliferation, including E2F target genes (Fig. 7d and S7C). Examples of genes where accessibility and nucleosome positioning changes coincided with gene activation include *Crabp1* and *Cdkn1c* (Supplementary Fig. 7d). In sum, these results indicate that ANP32E is a key regulator of developmental gene expression patterns in addition to its role in regulating H2A.Z localization and restricting genome-wide chromatin accessibility.

## Discussion

Here we demonstrate that ANP32E restricts genome-wide chromatin accessibility through regulation of H2A.Z accumulation and H2A.Z localization. In mouse fibroblasts lacking ANP32E, we found that heightened chromatin accessibility corresponded with increased H2A.Z (Fig. 2), and with differences in H2A.Z positioning at promoters (Fig. 3). We therefore define H2A.Z as a universal chromatin accessibility factor, and delineate three classes of promoters based on how H2A.Z is localized. Class Zero promoters lack H2A.Z, have poorly positioned nucleosomes, and are largely inaccessible. Class One promoters have H2A.Z at the +1 nucleosome, have moderately positioned nucleosomes, and have intermediate levels of accessibility. Class Two promoters have H2A.Z at both the +1 and −1 nucleosome positions, have well positioned nucleosomes flanking the NDR, and have the highest degree of chromatin accessibility. Consistent with this hierarchical organization strategy, loss of ANP32E caused the greatest increase in short chromatin fragments at promoters where H2A.Z expanded to both TSS flanking positions. These accessibility changes are dependent on H2A.Z levels (Figs. 4 and 5), and correspond with changes in SMARCA4 enrichment (Fig. 6). We also found that heightening of accessibility at TF-binding motifs corresponded with increased gene expression (Fig. 7). Thus, ANP32E abundance determines how genes are organized along the H2A.Z hierarchy. High levels of ANP32E drives promoters down the hierarchy to lower H2A.Z-positioning classes, and low levels of ANP32E allows promoters to rise to higher H2A.Z classes. Taken together, these results indicate that through global regulation of H2A.Z, ANP32E restricts TF-binding genome wide, and in doing so, controls widespread transcription levels (Fig. 7e).

Cognizant of the various reports that H2A.Z has a dual function as an activator and a silencer^4,13,22,53^, at the onset of this study we expected to find two classes of H2A.Z-marked genes, one class where H2A.Z expansion led to gene activation, and another where H2A.Z expansion led to silencing. However, when we assessed H2A.Z enrichment across two mouse embryonic cell types, we found that H2A.Z was frequently present at active genes, and H2A.Z was rarely present at silenced genes. Furthermore, H2A.Z-marked gene promoters that gained more H2A.Z in the absence of ANP32E, and became more accessible, tended to be up-regulated. Based on these results, we speculate that H2A.Z functions primarily as an activator. This view of H2A.Z aligns well with the vast majority of studies focused on H2A.Z transcriptional function, including early *Tetrahymena* studies describing H2A.Z presence in only the transcriptionally active macronucleus^54^, and studies describing H2A.Z as a semi-permissible or “labile” barrier to polymerase during gene activation^10,17,55,56^. To reconcile our findings with descriptions of H2A.Z functioning in gene silencing^3,4,22^, we favor a model where H2A.Z promotes activation in differentiated cells, but contributes to both activation and repression in pluripotent cells, perhaps through unknown cell-type specific mediators. Indeed, we did find evidence that H2A.Z corresponds with the silencing epigenetic mark H3K27me3 in mouse stem cells, suggesting that H2A.Z might function in gene silencing uniquely in stem cells. It will be important for future studies to test this concept and determine whether H2A.Z function may be cell-type or cell-state specific.

Our demonstration that H2A.Z promotes increased TF binding agrees with prior reports describing how H2A.Z functions in OCT4 and FOXA2 recruitment^4,5^. Our results are also consistent with studies describing how H2A.Z depletion leads to decreased nucleosome levels^4^ in mESCs. Several known characteristics of H2A.Z nucleosomes might account for region-specific differences in chromatin accessibility. For example, H2A.Z-containing nucleosomes may be more loosely wrapped than canonical nucleosomes^35^, and this might facilitate increased TF binding. Based on this model, TF binding might occur without the need to displace the underlying nucleosomes, and in this regard, chromatin at the TF-bound regions would harbor more weakly positioned nucleosomes. However, in our study we found that H2A.Z sites surrounding the TSS corresponded with more strongly positioning nucleosomes. Additionally, we found that increased accessibility occurred between, but not directly overlapping with H2A.Z sites. We also found that SMARCA4, the mammalian homolog for RSC, binds at the same locations where accessibility changes occur (over the NDR). Therefore, we favor a model in which H2A.Z-containing nucleosomes are the preferred substrate for nucleosome remodelers, and this preference leads to increased chromatin accessibility. We propose that increased nucleosome remodeling at H2A.Z sites leads to increased nucleosome repositioning, which uncovers TF-binding motifs and promotes increased gene expression. This model is in agreement with studies of chromatin remodelers in yeast and plants, which demonstrate that ISWI^15^, RSC^14^, and BRM^16^ prefer to remodel H2A.Z-containing nucleosomes. Although this mechanism has not yet been demonstrated in animals, SMARCA4 and H2A.Z do physically interact in mESCs^5^. Additionally, increased nucleosome turnover might occur as a function of remodeling, perhaps allowing for better TF binding. In this scenario, H2A.Z installation would increase without increases in total residency, possibly due to H2A.Z saturation. This mechanism might account for why some of the H2A.Z enrichment changes we observed were less dramatic than the observed chromatin accessibility changes. While data from this study indicate that ANP32E inhibits SMARCA4 enrichment, the NucleoATAC methods we used are unable to distinguish between impacts on nucleosome stability and impacts on nucleosome remodeling. Therefore, future biochemical studies are necessary to test whether the activity of mammalian remodelers are indeed influenced by H2A.Z and ANP32E. In addition to the SMARCA4- containing BRG complex, mammals harbor several other nucleosome remodelers within the SWI/SNF and ISWI families^51,57^. Thus, chromatin changes may have occurred though the combined impacts of several remodeling factors. It will be important for future studies to test whether there is functional conservation in nucleosome remodeling at H2A.Z sites and to determine the role of ANP32E in regulation of these additional remodelers.

In the present study, we found that ANP32E loss led to H2A.Z accumulation mostly at the −1 nucleosome position relative to the TSS. While it is possible that this occurred through disruption of targeted H2A.Z eviction, no mechanism for ANP32E targeting has been described. On the contrary, targeted H2A.Z installation is known to occur specifically at the NDRs and at the +1 nucleosome position^11,36,37,58^. Therefore, rather than invoking H2A.Z eviction, we favor a simpler explanation for how H2A.Z accumulated at the −1 position. We propose that H2A.Z installation occurs in a targeted and hierarchical manner (Fig. 7e), whereby H2A.Z is installed primarily at the +1 nucleosome, and at sites where H2A.Z is already present, ancillary H2A.Z is installed at the −1 position. Therefore, at promoters where H2A.Z installation is high, H2A.Z resides at both TSS flanking positions, and at moderately targeted promoters, H2A.Z resides only at the +1 position. Based on this concept, ANP32E might sequester free H2A.Z to partially inhibit installation, leading to H2A.Z enrichment only at the +1 nucleosome, and not the −1 nucleosome position. Loss of ANP32E would therefore allow for more H2A.Z availability, and ancillary H2A.Z installation would occur at the −1 nucleosome at moderately targeted promoters. This model fits well with prior studies reporting higher levels of H2A.Z at the +1 nucleosome position in mammals^19,33,59^, as well as studies in Drosophila and Arabidopsis where homologs of H2A.Z are absent from the −1 nucleosome position^13,60,61^. Notably, we cannot exclude the possibility that ANP32E functions specifically to evict H2A.Z at the −1 nucleosome. Thus, additional studies are necessary to determine how H2A.Z targeting occurs, to identify the factors that direct H2A.Z accumulation preferentially at the +1 and −1 nucleosome positions, and to assess the in vivo ability of ANP32E to evict H2A.Z nucleosomes.

We found that loss of ANP32E in MEFs resulted in dramatic gene expression changes (Fig. 7). Transcription increased at genes where small non-nucleosomal DNA fragments accumulated, and expression decreased where these fragments were lacking. These small non-nucleosomal fragments occurred mostly at promoters where H2A.Z levels increased and nucleosome positioning strengthened (Fig. 3e, f and Supplementary Fig. 6c–e). In addition, we identified protected footprinting sites at motifs for known regulators of differentiation, including SP1 and NF-Y (Fig. 5). Importantly, knockdown of H2A.Z in ANP32E-deficient cells caused reversal in protection for many of these footprints, and rescued the increased accessibility measured at TSS regions (Figs. 4 and 5d). These results indicate that H2A.Z accumulation is an upstream driver of heightened chromatin accessibility, leading to increased TF binding and activation of gene expression. While it is possible that some H2A.Z changes also occurred downstream of transcriptional impacts, we did not find clear evidence supporting this in our study. In GSEA tests, we find that genes involved in developmental differentiation became activated and genes involved in cell cycle progression became silenced when ANP32E was lacking (Supplementary Fig. 7). Furthermore, when we performed independent GO analysis on different classes of H2A.Z-marked promoters, we again identified pathways associated with developmental differentiation for genes where H2A.Z increased (Supplementary Fig. 3d). The remarkable consistency between biological pathways identified for H2A.Z localization changes and transcriptional changes suggests that ANP32E might be a critical regulator of cell-state transition. Our results imply that high levels of ANP32E may help to maintain proliferation and self-renewal, while low levels of ANP32E may permit cellular transitions and differentiation. This concept aligns well with recent studies of ANP32E in breast cancer^62^, where high *ANP32E* expression corresponded with activation of *E2F1*, more efficient G1/S transition, and cellular proliferation. Thus, it will be critical for future studies to investigate how levels of H2A.Z and ANP32E are balanced in a cell-type-specific manner, and how these factors might regulate TF binding, cell cycle progression, and cell-state transitions.

Several reports suggest that the precise regulation of H2A.Z positioning might be critical for preventing cancer. Amplified expression of H2A.Z has been associated with poor prognosis for several cancer types, including glioma, melanoma, and breast cancer^63^. Studies of prostate cancer have found that H2A.Z-positioning flanking de novo NDRs occurs in association with increased oncogene expression^64,65^, and in Madin-Darby Canine Kidney cells, differences in H2A.Z-positioning flanking the TSS have been correlated with epithelial-to-mesenchymal transition^66^. Recent studies have also demonstrated that increased *ANP32E* expression in breast cancer is associated with tumor growth and proliferation^62^, perhaps occurring through the same mechanisms of H2A.Z regulation that we describe here. With consideration of these previously published results, our study suggests that precise control of ANP32E levels and H2A.Z positioning may be critical for preventing carcinogenesis. Thus, it will be important for future studies to investigate the mechanisms described here in the context of human diseases, including cancer.

## Methods

### Cell culture

Primary MEFs were from WT and *Anp32e*^−*/*−^ embryos and graciously donated to us by Dr. Ali Hamiche, at the Institut de Génétique et de Biologie Moléculaire et Cellulaire, Cedex, France. MEFs were cultured in Dulbeccos modified Eagle’s medium (Thermo 11995073) containing 10% fetal bovine serum and antibiotics. All assays were performed in parallel at passage numbers below 8. Approximately 200,000 cells were seeded on 10 cm plates 1 day prior to RNA isolation or ATAC library preparation. Approximately 100,000 cells were collected for CUT&Tag samples.

### H2A.Z siRNA knockdown

*Anp32e*^−/−^ MEFs were transfected using Lipofectamine RNAiMax (Thermo) with siRNAs, either non-targeting pool (Dharmacon D-001810-10-05) or a pool of 4 H2A.Z siRNAs (Dharmacon J-063612-10-0002, J-042994-10-0002, J-063612-09-0002, J-042994-09-0002). In all, 145 pmol siRNA pool was used for each 10 cm plate transfection, and cells were collected 48 h post transfection. H2A.Z (Active Motif 39113) and beta-Actin (Sigma A5441) antibodies were used for western blotting. Original scans of western blots are included as a Supplementary Source File (Supplementary Fig. 8). Cell lysates were from the same siRNA transfections used for western blot and ATAC-seq experiments.

### Genomics methods

Chromatin accessibility was measured using the Assay for Transposase-Accessible Chromatin combined with high-throughput sequencing (ATAC-Seq), and gene expression levels were assessed using high-throughput sequencing of RNA (RNA-Seq). Data were compared with datasets from studies which utilized chromatin immuno-precipitation sequencing (ChIP-Seq), but no new ChIP-Seq datasets were generated from this study. Data from numerous published studies were utilized^22,30,67–69^ (Supplementary Table 1). ATAC-Seq libraries were prepared using the Illumina Nextera DNA Flex Library Prep Kit (cat: 20018704) according to the standard methods. RNA-Seq libraries were prepared using the TruSeq Stranded Total RNA Library Prep Kit (cat: 20020598), according to the standard methods, and total RNA was purified using the Zymo Research Direct-zol RNA Miniprep kit (cat: R2051). Libraries were sequenced in 150 bp paired-end sequencing mode on an Illumina NextSeq550 high-throughput DNA Sequencer. CUT&Tag^48^ libraries were generated using approximately 100,000 cells. Cells were incubated with Convanavalin A-coated magnetic beads (Bang Laboratories, BP531), and then primary antibodies were added to incubate overnight at 4 °C. After incubation, secondary antibodies (incubated at RT for 1 h) and pA-Tn5 adapter complex (incubated at RT for 1 h) were added. Then, tagmentation took place for 1 h, and DNAs were extracted using phenol–chloroform–isoamyl alcohol. Libraries were prepared using NEBNext HiFi 2XPCR Master mix and cleaned up using Ampure XP beads (Beckman Coulter A63880). The following antibodies were used: Thermo PA542860 for ANP32E, Abcam ab110641 for SMARCA4, Santa Cruz sc-17824X for SP1, rabbit anti-mouse IgG antibody, and guinea pig anti-rabbit secondary antibody (antibodies online ABIN101961). CUT&Tag libraries were prepared using NEBNext HiFi 2XPCR Master mix, cleaned up using 1.1 volume Ampure XP beads, and pooled together for paired-end sequencing. Sequencing data deposited at NIH GEO Datasets: GSE145705.

### Bioinformatics analysis

*Alignment, normalization, and peak calling*: For ChIP-seq, CUT&Tag, ATAC-seq, fastq files were aligned to mouse genome (Mm10) using Bowtie2, and then PCR duplicates were removed using Picard. Read count normalization was performed on alignment files to account for sequencing depth differences, and genome browser tracks in bigwig format were generated from merged replicates using deeptools bamCoverage. Peak calling was performed using MACS2 (setting for H2A.Z ChIP: -f BAMPE–SPMR–nomodel -B–broad; for ATAC-seq: -f BAMPE–SPMR–nomodel -B). Mitochondrial peaks and peaks overlapping blacklisted regions were filtered out for downstream analysis. Intersection of peaks were performed using bedtools intersect, with intersecting peaks called as those with any amount of overlap, and venn diagrams of peak numbers were generated using R package eulerr. Scoring differential ATAC-Seq peaks was performed by DiffBind using union ATAC peaks of WT MEFs and *Anp32e*^−/−^ MEFs and triplicates of ATAC-seq with default setting. Conversion between bigwig and bedgraph took place using ucsc-binary-utilities, and data track visualization occurred using IGV.

*Partitioning of promoters based on H2A.Z change*: Normalized wild type and *Anp32e*^−*/*−^ H2A.Z ChIP-seq bigwig file were used to generate average scores over 500 bp window flanking both sides of TSS using deeptools. Then log2FC of H2A.Z was calculated using H2A.Z average scores for both sides of TSS. Promoters with increased H2A.Z at −1 nucleosome are defined as log2FC values in top 25% at −1 nucleosome and bottom 75% at +1 nucleosome. Promoters with increased H2A.Z at +1 nucleosome are defined as log2FC values in top 25% at +1 nucleosome and bottom 75% at −1 nucleosome.

*H2A.Z hierarchical positioning*: Within deeptools, normalized wild-type H2A.Z ChIP-seq bigwig files were used to generate average scores from −600 to −100 bp (−1 nucleosome side) and 100–600 bp (+1 nucleosome side) windows around TSS regions. The top 25% all H2A.Z average scores on both sides of TSSs were then used as the threshold value to partition promoters into different H2A.Z hierarchy categories (“Class Zero”, “Class One”, and “Class Two”) in R. For example, when average H2A.Z enrichment was scored in the top 25% on both sides of the TSS, this was classified as “Class Two” having H2A.Z at both −1 and +1 nucleosome position. To compare difference of H2A.Z hierarchical positioning between WT and *Anp32e*^−*/*−^ MEFs, threshold values established in WT were used. To compare mESCs with MEFs, threshold values established from mESC data were used. Partitioning of promoters occurred in R.

*TF footprinting and nucleosome positioning*: To identify TFs with differential footprints, narrowPeak files of wild type and *Anp32e*^−/−^ ATAC-seq and duplicates-removed bam files were used as input files for Rgt-Hint-ATAC (–atac-seq–paired-end–organism=mm10). Nucleosome positioning profiles were generated using duplicates-removed merged ATAC-seq bam files around promoter regions (TSS ± 1 kb) with default setting of nucleoATAC. Genome browser tracks were generated converting nucleoatac_signal.smooth.bedgraph to bigwig format using bedGraphToBigWig.

*Differential gene expression of RNA-seq data*: RNA-sequencing fastq files were aligned to mouse genome (Mm10) using RNA-STAR. Read count normalization was performed on alignment files to account for sequencing depth differences, and genome browser tracks in bigwig format were generated using deeptools bamCoverage. A heatmap of spearman correlation using normalized reads across samples was generated using deeptools multibigwigsummary and R package pheatmap. Differential gene expression analysis was performed using Subread featureCounts and DESeq2 with default setting. Genes on X and Y chromosomes were excluded. Differentially increased genes were defined as log2FC > 0.5 and adjusted *p* < 0.05, and differentially decreased genes were defined as log2FC < −0.5 and adjusted *p* < 0.05.

*GSEA and GO*: GSEA of gene expression between WT and *Anp32e*^−*/*−^ MEFs was performed on reads count table generated by featureCounts using default setting of GSEA (broad institute). GO analysis of differentially increased and decreased genes was performed using PANTHER. GO of TSS from different H2A.Z hierarchical positioning was performed using GREAT^70^ with user input background datasets.

*Plotting*: Heatmaps and average aggregate plots were generated using deeptools plotHeatmap and plotProfile. All plots of promoters were oriented with the direction of gene transcription going from left to right. Heatmaps of spearman correlation values were generated using R pheatmap, and boxplots and volcano plots were generated using standard R. Fonts and labels were adjusted with Affinity Designer.

### Statistical methods

Comparisons between different groups in boxplots were performed using pairwise Wilcoxon rank-sum test and two-sided Student’s *t*-tests, with *p* values adjusted using the “Hochberg” method. Default statistical thresholds (adjusted *p* < 0.01) were applied as part of DESeq2 to identify differentially expressed genes in RNA-Seq analysis, unless otherwise stated. For correlational analysis, a Spearman correlation test was applied on complete datasets—regions where data were missing were excluded and *R*-values are indicated where applicable. GO analysis and GSEA were performed under default settings and adjusted *p* values or FDR values are indicated in the plot. Hypergeometric test was applied to compare overlapping H2A.Z peaks and ATAC peaks, and *p* values are indicated in the plot.

### Reporting summary

Further information on research design is available in the Nature Research Reporting Summary linked to this article.

## Supplementary information

Supplementary Information

Peer Review File

Reporting Summary

## Data Availability

The data that support this study are available from the corresponding author upon reasonable request. High-throughput sequencing data that support the findings of this study are available through the NIH GEO Database using the accession number GSE145705.

## References

[1] Ong C-T, Corces VG (2011). Enhancer function: new insights into the regulation of tissue-specific gene expression. Nat. Rev. Genet..

[2] Brunelle M (2015). The histone variant H2A.Z is an important regulator of enhancer activity. Nucleic Acids Res..

[3] Creyghton MP (2008). H2AZ is enriched at polycomb complex target genes in ES cells and is necessary for lineage commitment. Cell.

[4] Hu G (2013). H2A.Z facilitates access of active and repressive complexes to chromatin in embryonic stem cell self-renewal and differentiation. Cell Stem Cell.

[5] Li Z (2012). Foxa2 and H2A.Z mediate nucleosome depletion during embryonic stem cell differentiation. Cell.

[6] Klemm SL, Shipony Z, Greenleaf WJ (2019). Chromatin accessibility and the regulatory epigenome. Nat. Rev. Genet..

[7] Thurman RE (2012). The accessible chromatin landscape of the human genome. Nature.

[8] Donaghey J (2018). Genetic determinants and epigenetic effects of pioneer-factor occupancy. Nat. Genet..

[9] Zaret KS, Mango SE (2016). Pioneer transcription factors, chromatin dynamics, and cell fate control. Curr. Opin. Genet. Dev..

[10] Jin C, Felsenfeld G (2007). Nucleosome stability mediated by histone variants H3.3 and H2A.Z. Genes Dev..

[11] Jin C (2009). H3.3/H2A.Z double variant-containing nucleosomes mark ‘nucleosome-free regions’ of active promoters and other regulatory regions. Nat. Genet..

[12] Ramachandran S, Ahmad K, Henikoff S (2017). Transcription and remodeling produce asymmetrically unwrapped nucleosomal intermediates. Mol. Cell.

[13] Weber CM, Henikoff S (2014). Histone variants: dynamic punctuation in transcription. Genes Dev..

[14] Cakiroglu A (2019). Genome-wide reconstitution of chromatin transactions reveals that RSC preferentially disrupts H2A.Z-containing nucleosomes. Genome Res..

[15] Goldman JA, Garlick JD, Kingston RE (2010). Chromatin remodeling by Imitation Switch (ISWI) class ATP-dependent remodelers is stimulated by histone variant H2A.Z. J. Biol. Chem..

[16] Torres ES, Deal RB (2019). The histone variant H2A.Z and chromatin remodeler BRAHMA act coordinately and antagonistically to regulate transcription and nucleosome dynamics in Arabidopsis plant. J. Cell Mol. Biol..

[17] Weber CM, Ramachandran S, Henikoff S (2014). Nucleosomes are context-specific, H2A.Z-modulated barriers to RNA polymerase. Mol. Cell.

[18] Ku M (2012). H2A.Z landscapes and dual modifications in pluripotent and multipotent stem cells underlie complex genome regulatory functions. Genome Biol..

[19] Subramanian V (2013). H2A.Z acidic patch couples chromatin dynamics to regulation of gene expression programs during ESC differentiation. PLoS Genet.

[20] Sutcliffe EL (2009). Dynamic histone variant exchange accompanies gene induction in T cells. Mol. Cell. Biol..

[21] Weber CM, Henikoff JG, Henikoff S (2010). H2A.Z nucleosomes enriched over active genes are homotypic. Nat. Struct. Mol. Biol..

[22] Hsu C-C (2018). Gas41 links histone acetylation to H2A.Z deposition and maintenance of embryonic stem cell identity. Cell Discov..

[23] Bernstein BE (2006). A bivalent chromatin structure marks key developmental genes in embryonic stem. Cells Cell.

[24] Brahma S (2017). INO80 exchanges H2A.Z for H2A by translocating on DNA proximal to histone dimers. Nat. Commun..

[25] Choi J, Heo K, An W (2009). Cooperative action of TIP48 and TIP49 in H2A.Z exchange catalyzed by acetylation of nucleosomal H2A. Nucleic Acids Res..

[26] Kobor MS (2004). A protein complex containing the conserved Swi2/Snf2-related ATPase Swr1p deposits histone variant H2A.Z into euchromatin. PLoS Biol..

[27] Krogan NJ (2003). A Snf2 family ATPase complex required for recruitment of the histone H2A variant Htz1. Mol. Cell.

[28] Papamichos-Chronakis M, Watanabe S, Rando OJ, Peterson CL (2011). Global regulation of H2A.Z localization by the INO80 chromatin-remodeling enzyme is essential for genome integrity. Cell.

[29] Ruhl DD (2006). Purification of a human SRCAP complex that remodels chromatin by incorporating the histone variant H2A.Z into nucleosomes. Biochemistry.

[30] Obri A (2014). ANP32E is a histone chaperone that removes H2A.Z from chromatin. Nature.

[31] Murphy PJ, Wu SF, James CR, Wike CL, Cairns BR (2018). Placeholder nucleosomes underlie germline-to-embryo DNA methylation reprogramming. Cell.

[32] Reilly PT (2010). Generation and characterization of the Anp32e-deficient mouse. PLoS ONE.

[33] Mao Z (2014). Anp32e, a higher eukaryotic histone chaperone directs preferential recognition for H2A.Z. Cell Res..

[34] Barski A (2007). High-resolution profiling of histone methylations in the human genome. Cell.

[35] Talbert PB, Henikoff S (2010). Histone variants—ancient wrap artists of the epigenome. Nat. Rev. Mol. Cell Biol..

[36] Yen K, Vinayachandran V, Pugh BF (2013). SWR-C and INO80 chromatin remodelers recognize nucleosome-free regions near +1 nucleosomes. Cell.

[37] Ranjan A (2013). Nucleosome-free region dominates histone acetylation in targeting SWR1 to promoters for H2A.Z replacement. Cell.

[38] Sun L, Pierrakeas L, Li T, Luk E (2020). Thermosensitive nucleosome editing reveals the role of DNA sequence in targeted histone variant deposition. Cell Rep..

[39] Buenrostro JD, Giresi PG, Zaba LC, Chang HY, Greenleaf WJ (2013). Transposition of native chromatin for fast and sensitive epigenomic profiling of open chromatin, DNA-binding proteins and nucleosome position. Nat. Methods.

[40] Li Z (2019). Identification of transcription factor binding sites using ATAC-seq. Genome Biol..

[41] Gusmao EG, Allhoff M, Zenke M, Costa IG (2016). Analysis of computational footprinting methods for DNase sequencing experiments. Nat. Methods.

[42] Cavanaugh E, DiMario JX (2017). Sp3 controls fibroblast growth factor receptor 4 gene activity during myogenic differentiation. Gene.

[43] Gilmour J (2019). Robust hematopoietic specification requires the ubiquitous Sp1 and Sp3 transcription factors. Epigenet. Chromatin.

[44] Maity SN (2017). NF-Y (CBF) regulation in specific cell types and mouse models. Biochim. Biophys. Acta BBA Gene Regul. Mech..

[45] Parakati R, DiMario JX (2002). Sp1- and Sp3-mediated transcriptional regulation of the fibroblast growth factor receptor 1 gene in chicken skeletal muscle cells. J. Biol. Chem..

[46] Roder K, Wolf SS, Larkin KJ, Schweizer M (1999). Interaction between the two ubiquitously expressed transcription factors NF-Y and Sp1. Gene.

[47] Yamada K, Tanaka T, Miyamoto K, Noguchi T (2000). Sp family members and nuclear factor-Y cooperatively stimulate transcription from the rat pyruvate kinase M gene distal promoter region via their direct interactions. J. Biol. Chem..

[48] Kaya-Okur HS (2019). CUT&Tag for efficient epigenomic profiling of small samples and single cells. Nat. Commun..

[49] Nie Z (2000). A specificity and targeting subunit of a human SWI/SNF family-related chromatin-remodeling complex. Mol. Cell. Biol..

[50] Vierbuchen T (2017). AP-1 transcription factors and the BAF complex mediate signal-dependent enhancer selection. Mol. Cell.

[51] Kadoch C, Crabtree GR (2015). Mammalian SWI/SNF chromatin remodeling complexes and cancer: mechanistic insights gained from human genomics. Sci. Adv..

[52] Schep AN (2015). Structured nucleosome fingerprints enable high-resolution mapping of chromatin architecture within regulatory regions. Genome Res..

[53] Subramanian, V., Fields, P. A. & Boyer, L. A. H2A.Z: a molecular rheostat for transcriptional control. *F1000Prime Rep*. **7**, 01 (2015). 10.12703/P7-01 PMC4311278 25705384

[54] Allis CD, Chicoine LG, Richman R, Schulman IG (1985). Deposition-related histone acetylation in micronuclei of conjugating tetrahymena. Proc. Natl Acad. Sci. USA.

[55] Adam M, Robert F, Larochelle M, Gaudreau L (2001). H2A.Z is required for global chromatin integrity and for recruitment of RNA polymerase II under specific conditions. Mol. Cell. Biol..

[56] Hardy S (2009). The euchromatic and heterochromatic landscapes are shaped by antagonizing effects of transcription on H2A.Z deposition. PLoS Genet.

[57] Wilson BG (2014). Residual complexes containing SMARCA2 (BRM) underlie the oncogenic drive of SMARCA4 (BRG1) mutation. Mol. Cell. Biol..

[58] Bagchi DN, Battenhouse AM, Park D, Iyer VR (2020). The histone variant H2A.Z in yeast is almost exclusively incorporated into the +1 nucleosome in the direction of transcription. Nucleic Acids Res.

[59] Rege M (2015). Chromatin dynamics and the RNA exosome function in concert to regulate transcriptional homeostasis. Cell Rep..

[60] Mavrich TN (2008). Nucleosome organization in the Drosophila genome. Nature.

[61] Zilberman D, Coleman-Derr D, Ballinger T, Henikoff S (2008). Histone H2A.Z and DNA methylation are mutually antagonistic chromatin marks. Nature.

[62] Xiong Z (2018). ANP32E induces tumorigenesis of triple-negative breast cancer cells by upregulating E2F1. Mol. Oncol..

[63] Vardabasso C (2014). Histone variants: emerging players in cancer biology. Cell. Mol. Life Sci..

[64] Valdés-Mora F (2012). Acetylation of H2A.Z is a key epigenetic modification associated with gene deregulation and epigenetic remodeling in cancer. Genome Res.

[65] Valdés-Mora F (2017). Acetylated histone variant H2A.Z is involved in the activation of neo-enhancers in prostate cancer. Nat. Commun..

[66] Domaschenz R, Kurscheid S, Nekrasov M, Han S, Tremethick DJ (2017). The histone variant H2A.Z is a master regulator of the epithelial-mesenchymal transition. Cell Rep..

[67] ENCODE Project Consortium. (2012). An integrated encyclopedia of DNA elements in the human genome. Nature.

[68] Juric I (2019). MAPS: model-based analysis of long-range chromatin interactions from PLAC-seq and HiChIP experiments. PLoS Comput. Biol..

[69] Xie W (2017). RNF40 regulates gene expression in an epigenetic context-dependent manner. Genome Biol..

[70] McLean CY (2010). GREAT improves functional interpretation of cis-regulatory regions. Nat. Biotechnol..

