# Supplementary material for: Genome-wide chromatin accessibility is restricted by ANP32E
Source: Nat Commun. 2020 Oct 8;11:5063. doi: 10.1038/s41467-020-18821-x (PMC7546623; doi:10.1038/s41467-020-18821-x)
Supplement: Supplementary file 1 — Supplementary Information [file 41467_2020_18821_MOESM1_ESM.pdf]

Supplemental Figure 1

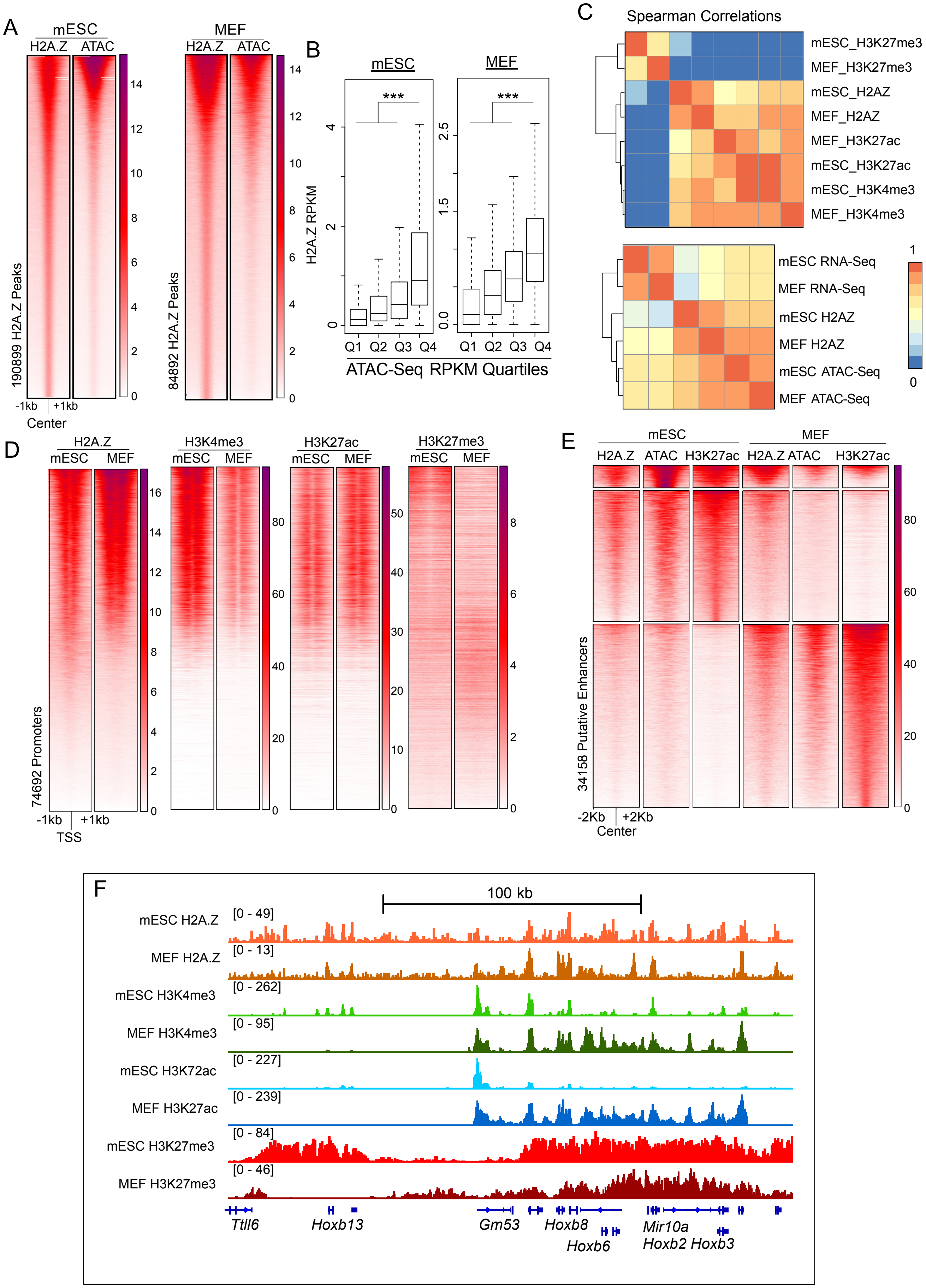

Supplemental Figure 1. Association of H2A.Z and chromatin accessibility.

(A) Heatmaps of normalized H2A.Z enrichment and chromatin accessibility signals at H2A.Z peaks showing strong correlation of H2A.Z and chromatin accessibility. H2A.Z peak regions (peak center ± 1kb) are used to generate heatmaps.
(B) Boxplots of H2A.Z enrichment RPKM values for chromatin accessibility quartiles showing that most accessible regions have higher H2A.Z levels. Randomly sampled regions (300bp, n=50,000) are used for plotting. (boxes = interquartile ranges, middles = medians, whiskers = 1.5X the interquartile range, adjusted p-values from pairwise two-sided Wilcoxon rank sum test, and *** indicates $p<0.0001$)
(C) Clustering of pairwise Spearman correlation values of several histone modifications and H2A.Z enrichment (top), as well as RNA, chromatin accessibility (ATAC) and H2A.Z enrichment (bottom) for mESCs and MEFs, displayed as heatmaps.
(D) Heatmaps comparing enrichment signals of H2A.Z, H3K4me3, H3K27ac and H3K27me3 at promoter regions (TSSs ± 1kb) in mESCs and MEFs. All heatmaps were generated using the same ordered regions ranked by decreasing H2A.Z levels in mESCs and MEFs.
(E) K-means clustering of heatmaps showing that H2A.Z correlates with chromatin accessibility at putative enhancers. Putative enhancers are defined as a union of H3K27ac-occupied regions more than 2kb away from promoters in mESCs and MEFs.
(F) Genome browser snapshots of the *Hoxb* locus showing H2A.Z enrichment and various epigenetic marks.

Supplemental Figure 2

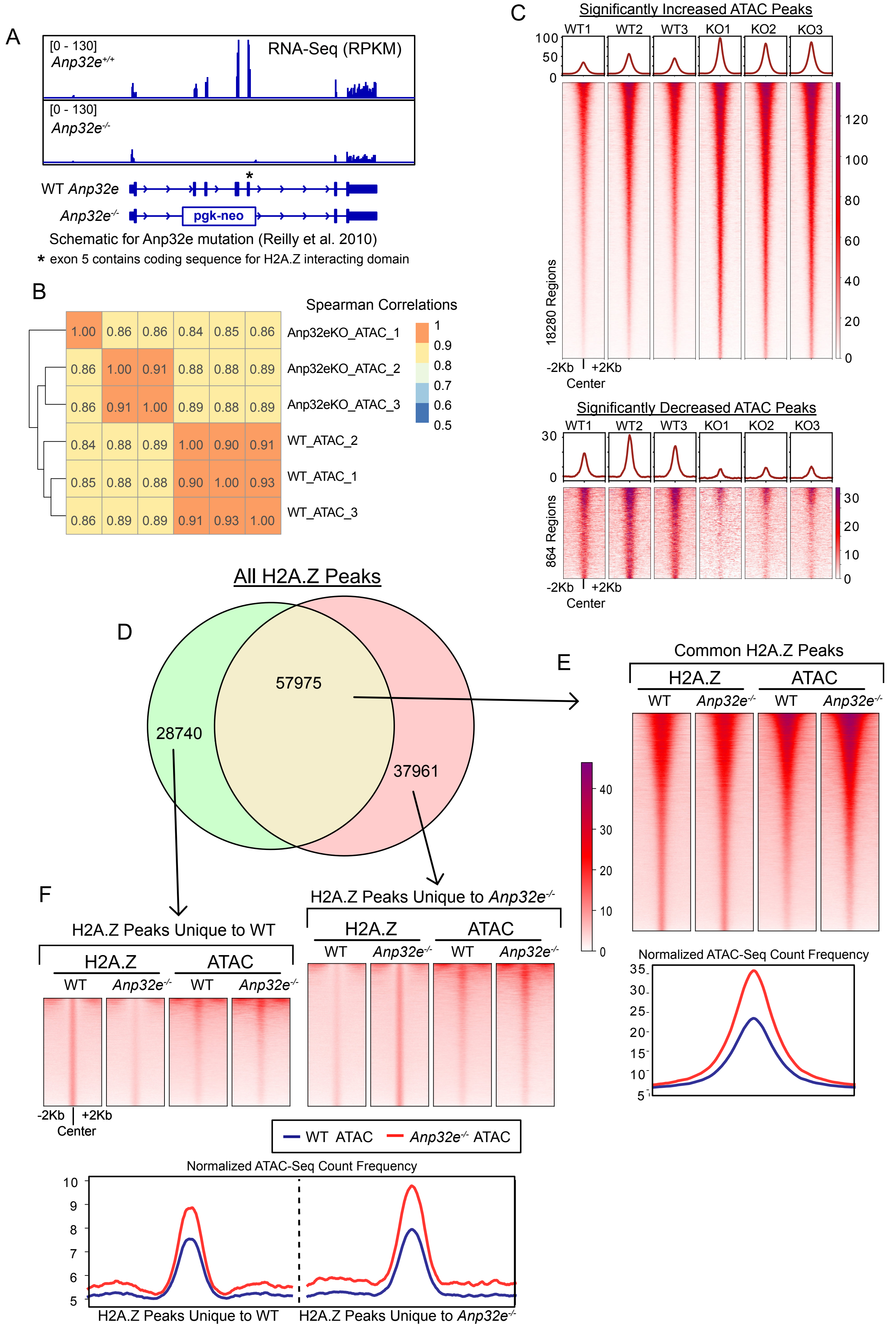

Supplemental Figure 2. Loss of ANP32E impacts chromatin patterns.

(A) Genome browser snapshot of RNA-seq signals at *Anp32e* locus confirms deletion of *Anp32e* coding sequence in the *Anp32e*$^{-/-}$ MEFs.
(B) A heatmap presentation of pairwise Spearman correlation values showing high correlation among chromatin accessibility replicates.
(C) Heatmaps of ATAC-seq replicates at differentially increased ATAC peaks (log2FC > 0.5 & adjusted p-value < 0.05, n=18,280) and differentially decreased ATAC peaks (log2FC < -0.5 & adjusted p-value < 0.05, n=864). Differential peaks were identified by DiffBind.
(D) Venn diagram showing overlap of WT and *Anp32e*$^{-/-}$ MEFs H2A.Z enrichment peaks. Numbers of H2A.Z peaks are shown in the plot.
(E) Heatmaps of H2A.Z enrichment and chromatin accessibility (top), and aggregate plots of chromatin accessibility (bottom) show increased chromatin accessibility in *Anp32e*$^{-/-}$ compared to WT MEFs at H2A.Z peaks common to WT and *Anp32e*$^{-/-}$ conditions (n=57,975).
(F) Heatmaps of H2A.Z enrichment and chromatin accessibility (top), and aggregate plots of chromatin accessibility (bottom) show increased chromatin accessibility in *Anp32e*$^{-/-}$ compared to WT MEFs at H2A.Z peaks unique to WT (n=28,740, left) and unique to *Anp32e*$^{-/-}$ (n=37,961, right).

Supplemental Figure 3

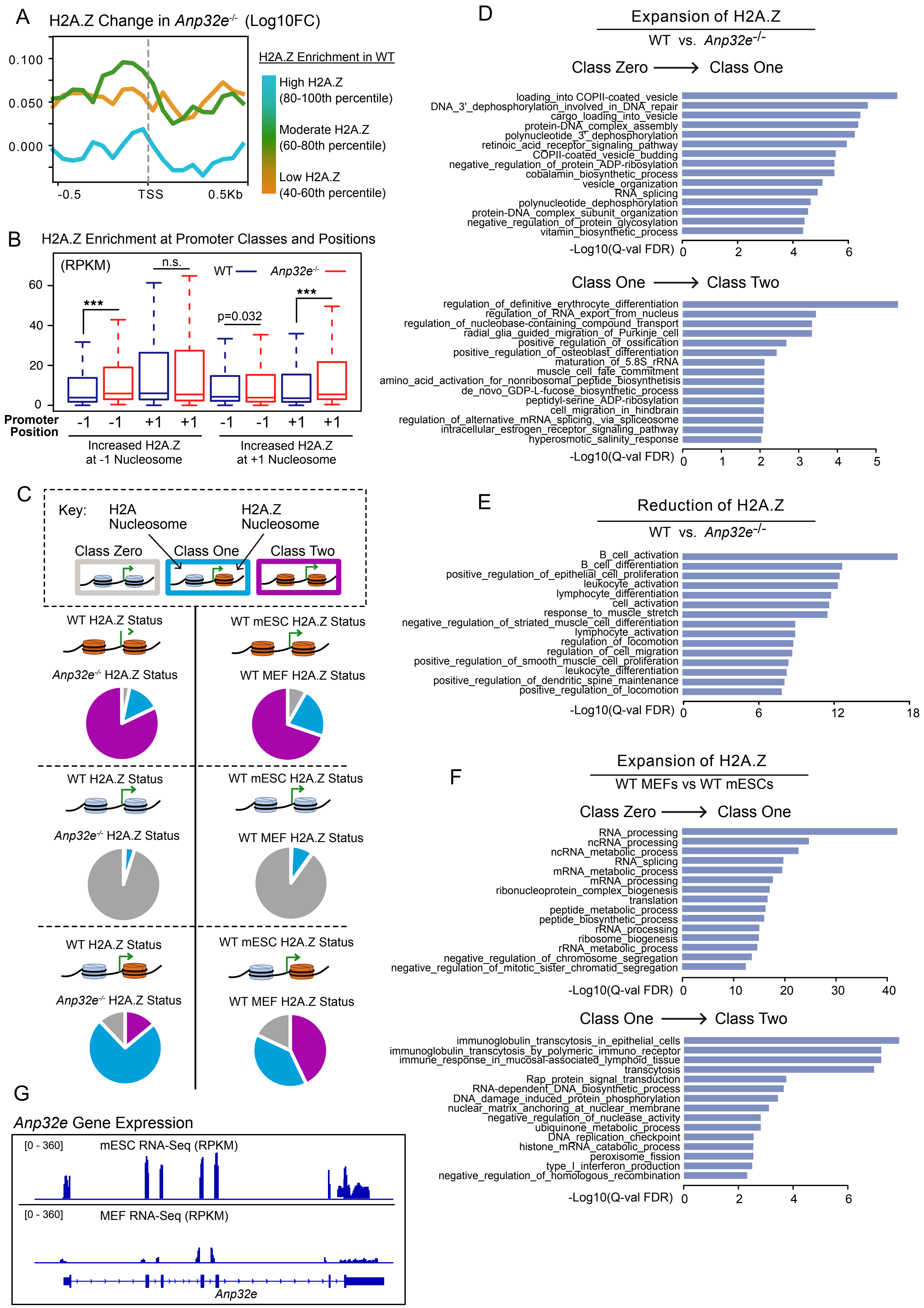

Supplemental Figure 3. Positional hierarchy defines H2A.Z patterns at promoters.

(A) Aggregate profile plot of log10FC H2A.Z level in Anp32e$^{-/-}$ MEFs at selected percentile of promoters. Percentile of all promoters are ranked by H2A.Z level in WT MEFs. The dotted line indicates location of TSSs.
(B) Boxplot of H2A.Z enrichment in WT MEFs to Anp32e$^{-/-}$ MEFs at promoters where H2A.Z increases at -1 nucleosome in Anp32e$^{-/-}$ MEFs, and promoters where H2A.Z increases at +1 nucleosome in Anp32e$^{-/-}$ MEFs. (n=74,692 total promoters, adjusted p-values from a two-sided Student's t-test, boxes = interquartile ranges, middles = medians, whiskers = 1.5X the interquartile range, *** indicates p<0.0001)
(C) Changes of H2A.Z status comparing WT MEFs to Anp32e$^{-/-}$ MEFs (left) or WT mESCs (right). Initial H2A.Z positioning status (either in WT MEF or WT mESCs) is indicated by the nucleosome cartoon depicted within each panel, and how H2A.Z positioning changed is indicated by the accompanying pie charts. Color key is included such that Class Zero promoters are grey, Class One promoters are blue, and Class Two promoters are purple.
(D) Enriched gene ontology terms for promoter where H2A.Z expands from WT MEFs to *Anp32e*$^{-/-}$ MEFs (Class Zero to Class One, and Class One to Class Two). Classes are depicted in Figure S3C, and the adjusted p-value for gene ontology terms are indicated.
(E) Enriched gene ontology terms as in panel D for promoter where H2A.Z is reduced from WT MEFs to *Anp32e*$^{-/-}$ MEFs.
(F) Enriched gene ontology terms as in panel D for promoter where H2A.Z expands from WT mESCs to WT MEFs *Anp32e*$^{-/-}$ MEFs.
(G) A genome browser snapshot of normalized RNA expression at the *Anp32e* locus showing higher *Anp32e* expression level in mESCs compared to MEFs.

Supplemental Figure 4

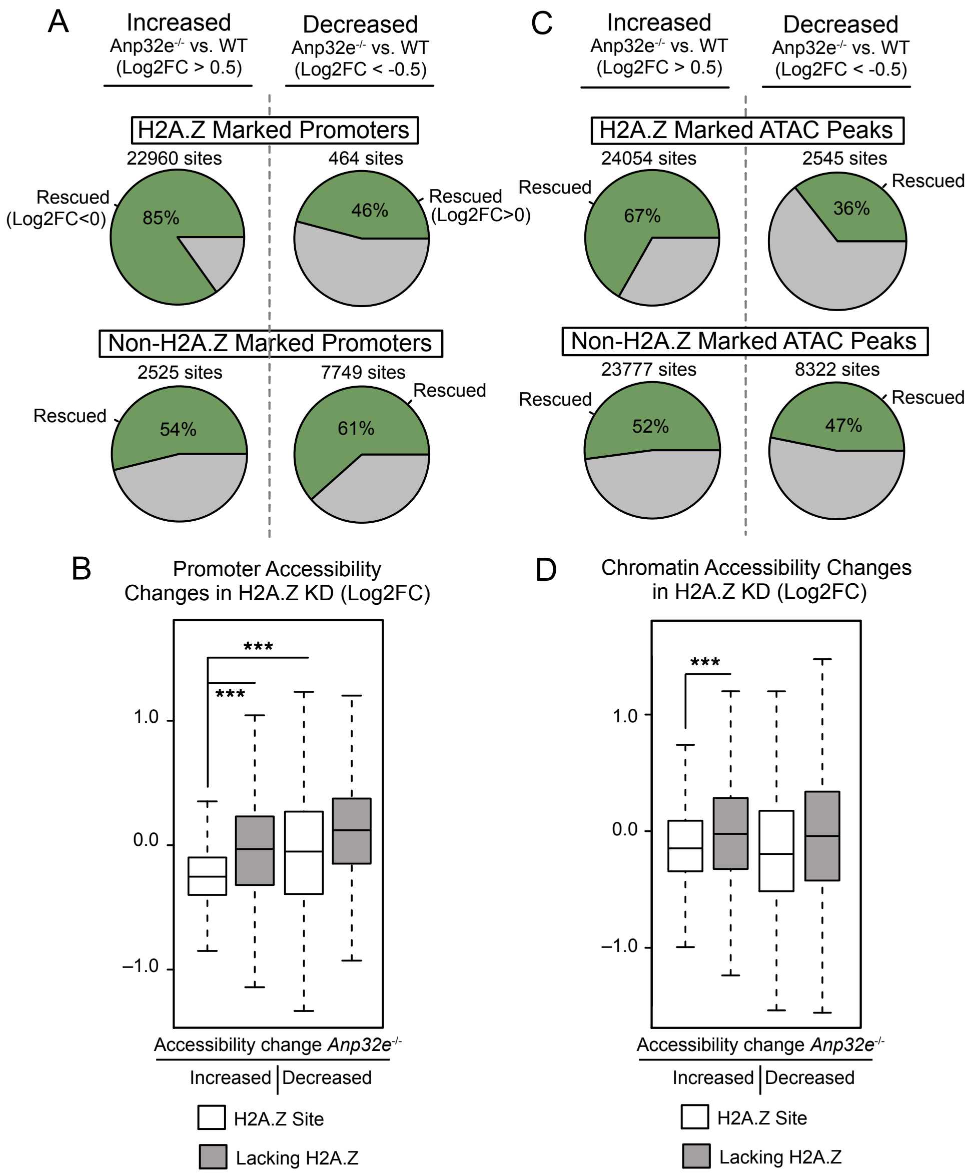

Supplemental Figure 4. Impacts of ANP32E loss are dependent on H2A.Z.

(A) Pie charts of changes in chromatin accessibility at H2A.Z marked promoter regions (TSS ± 1kb, top panel) and non-H2A.Z marked promoter regions (TSS ± 1kb, bottom panel) after H2A.Z knockdown. Increased and decreased promoters are defined based on 0.5 log2FC of *Anp32e*$^{-/-}$ ATAC-Seq versus WT ATAC-Seq, and "Rescued" is defined any change that occurred in an opposite direction in *Anp32e*$^{-/-}$ H2A.Z siRNA versus non-targeting control siRNA ATAC-Seq. The number of promoters represented by the pie chart, and the rescue percentage are labelled in each pie chart.
(B) Boxplot of log2FC of *Anp32e*$^{-/-}$ H2A.Z siRNA versus non-targeting control siRNA ATAC-Seq at promoter regions. H2A.Z marked promoters are represented in unfilled boxes, and non-H2A.Z marked promoters are represented in filled boxes. (n=74,692 total promoters, adjusted p-values from a two-sided Student's t-test, boxes = interquartile ranges, middles = medians, whiskers = 1.5X the interquartile range, *** indicates p<0.0001)
(C) Pie charts of changes of chromatin accessibility at H2A.Z marked ATAC peaks (top panel) and non-H2A.Z marked ATAC peaks (bottom panel) after H2A.Z knockdown. Increased and Decreased promoters are defined based on 0.5 log2FC of *Anp32e*$^{-/-}$ ATAC-Seq versus WT ATAC-Seq, and rescued are defined based on 0 log2FC of *Anp32e*$^{-/-}$ H2A.Z siRNA versus NT control siRNA ATAC-Seq. The number of promoters represented by the pie chart, and the percentage of rescue are labelled in each pie chart.
(D) Boxplot of log2FC of *Anp32e*$^{-/-}$ H2A.Z siRNA versus non-targeting control siRNA ATAC-Seq at ATAC peaks. H2A.Z marked ATAC peaks are represented in unfilled boxes, and non-H2A.Z marked ATAC peaks are represented in filled boxes. (n=102,911 ATAC peaks, adjusted p-values from a two-sided Student's t-test, boxes = interquartile ranges, middles = medians, whiskers = 1.5X the interquartile range, *** indicates p<0.0001)

Supplemental Figure 5

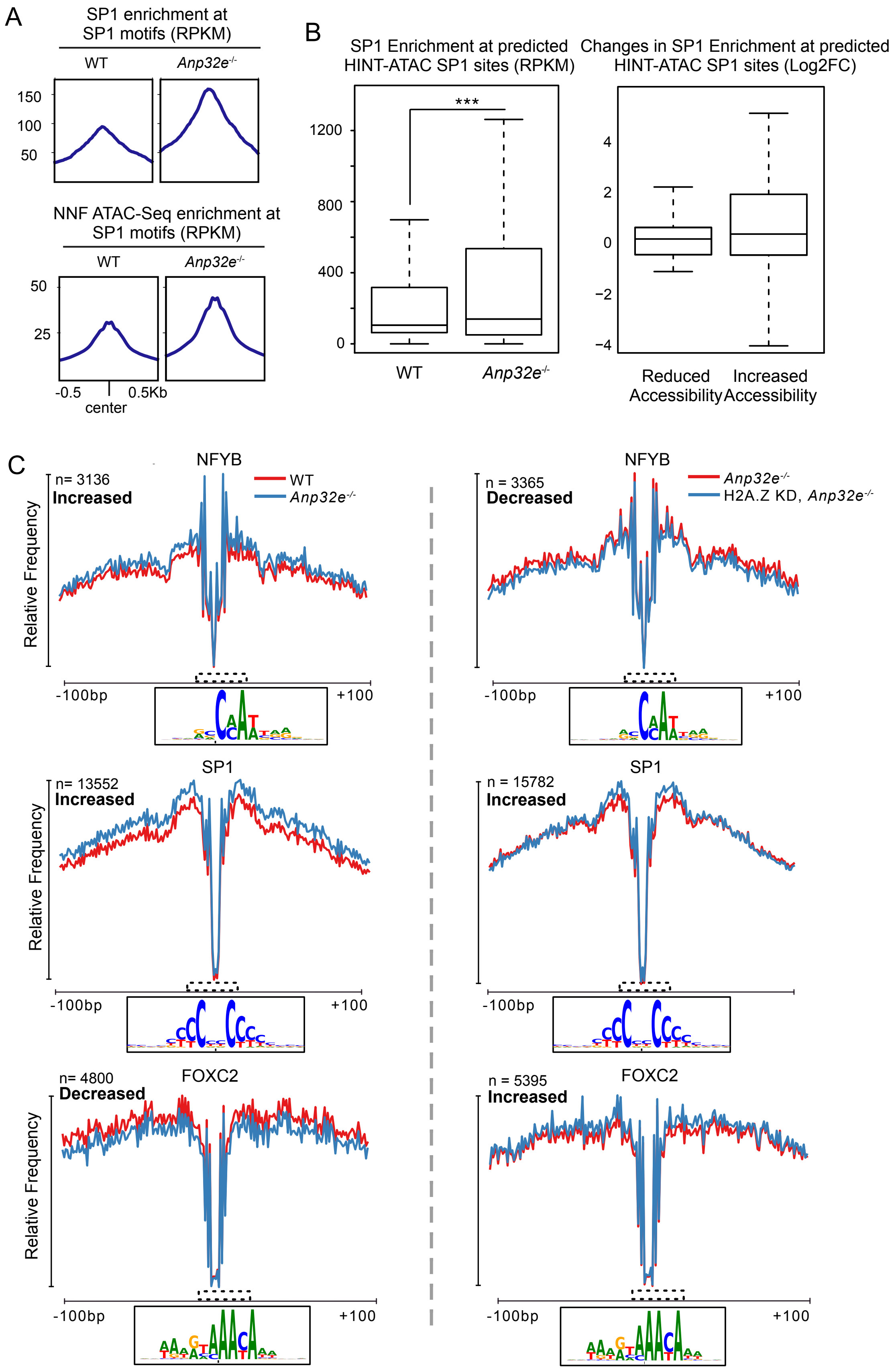

Supplemental Figure 5. Loss of ANP32E impacts TF binding.

(A) Aggregate profile plots of SP1 CUT&Tag and non-nucleosome fragment (NNF) of ATAC-Seq at all putative SP1 motifs (n=68,479). Putative SP1 motifs in the mm10 genome were identified by Homer.
(B) Boxplot of RPKM (left) and log2FC (right) for SP1 enrichment at strong putative HINT-ATAC SP1 sites. HINT-ATAC SP1 sites were used to calculate enrichment score of SP1 in WT MEFs and *Anp32e*$^{-/-}$ MEFs, and the 90th percentile of SP1-enriched sites was used for analysis as these were considered as strong putative SP1 binding sites (n=1159, adjusted p-values from a two-sided Student's t-test, boxes = interquartile ranges, middles = medians, whiskers = 1.5X the interquartile range, *** indicates $p<0.0001$)
(C) Average cleavage profiles of NFYB, SP1, and FOXC2 motifs identified by HINT-ATAC comparing *Anp32e*$^{-/-}$ ATAC-Seq and WT ATAC-Seq (left panels), and *Anp32e*$^{-/-}$ H2A.Z siRNA ATAC-Seq and *Anp32e*$^{-/-}$ NT control siRNA ATAC-Seq (right panels). A magnified view of the sequence motif (dashed box) for each TF is shown below profile plot.

Supplemental Figure 6

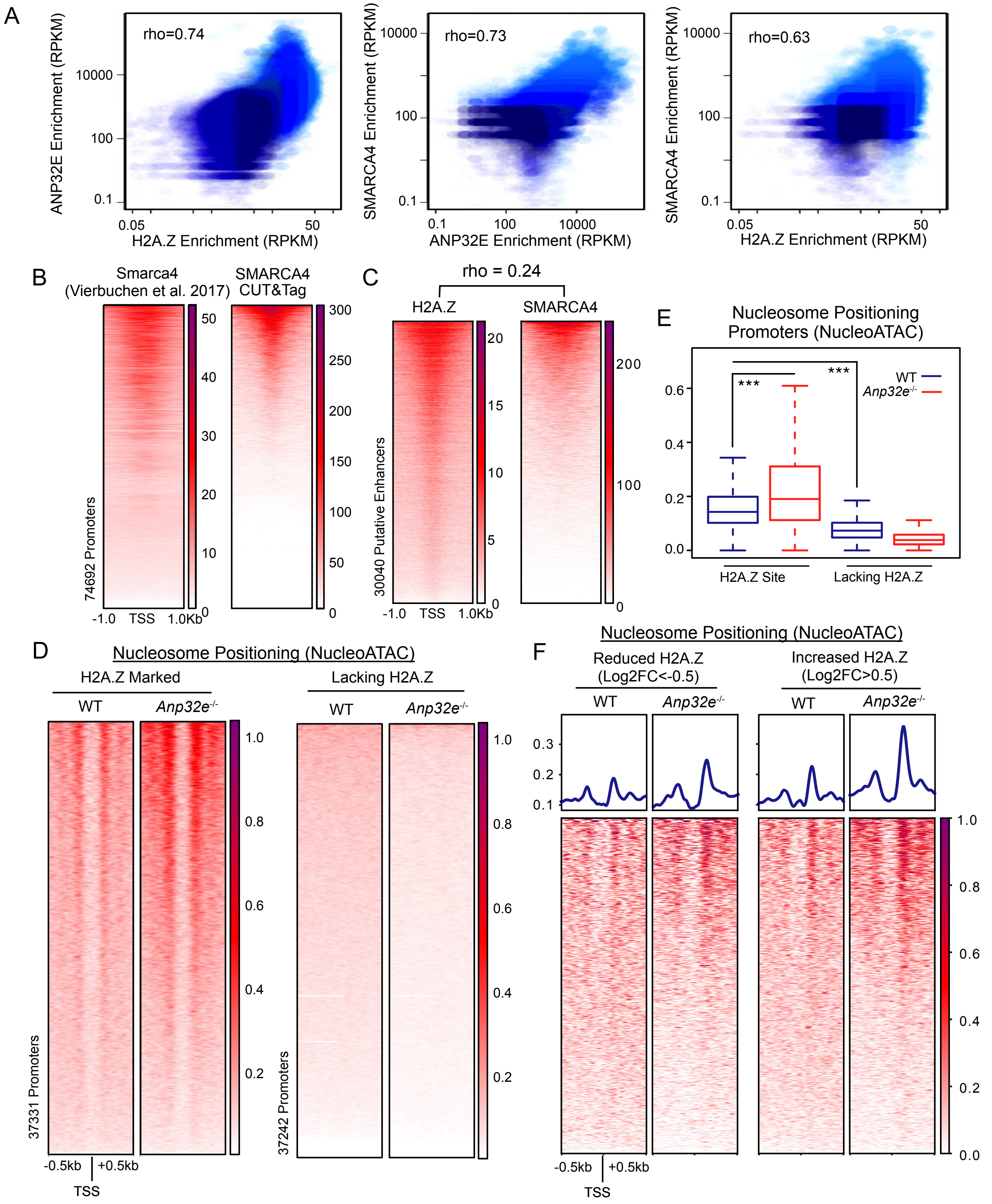

Supplemental Figure 6. Nucleosome remodeling defects occur upon ANP32E loss.

(A) Pairwise scatterplot of ANP32E, H2A.Z and SMARCA4 enrichment signal at promoter regions (TSS ± 1kb). Spearman's correlation coefficient is indicated in each scatterplot, and color intensity represents density of points plotted.
(B) Heatmap showing SMARCA4 CUT&Tag profile overlaps with SMARCA4 ChIP-Seq profile at promoter regions.
(C) Heatmap showing that H2A.Z enrichment partially overlaps with SMARCA4 enrichment at putative enhancers. Pairwise Spearman correlation rho value is indicated.
(D) Heatmaps of nucleosome positioning identified by NucleoATAC in WT MEFs and *Anp32e*$^{-/-}$ MEFs at H2A.Z marked promoters and non-H2A.Z marked promoters.
(E) Boxplot of nucleosome positioning in WT MEFs and *Anp32e*$^{-/-}$ MEFs at H2A.Z marked promoters and non-H2A.Z marked promoters. (n=74,692 total promoters, boxes = interquartile ranges, middles = medians, whiskers = 1.5X the interquartile range, adjusted p-values from pairwise two-sided Wilcoxon rank sum test, and *** indicates $p<0.0001$)
(F) Heatmaps showing changes in nucleosome positioning identified by NucleoATAC at H2A.Z marked promoters with reduced H2A.Z level (log2FC < -0.5, n=2,304) and increased H2A.Z level (log2FC > 0.5, n=3,454) in WT MEFs and *Anp32e*$^{-/-}$ MEFs.

Supplemental Figure 7

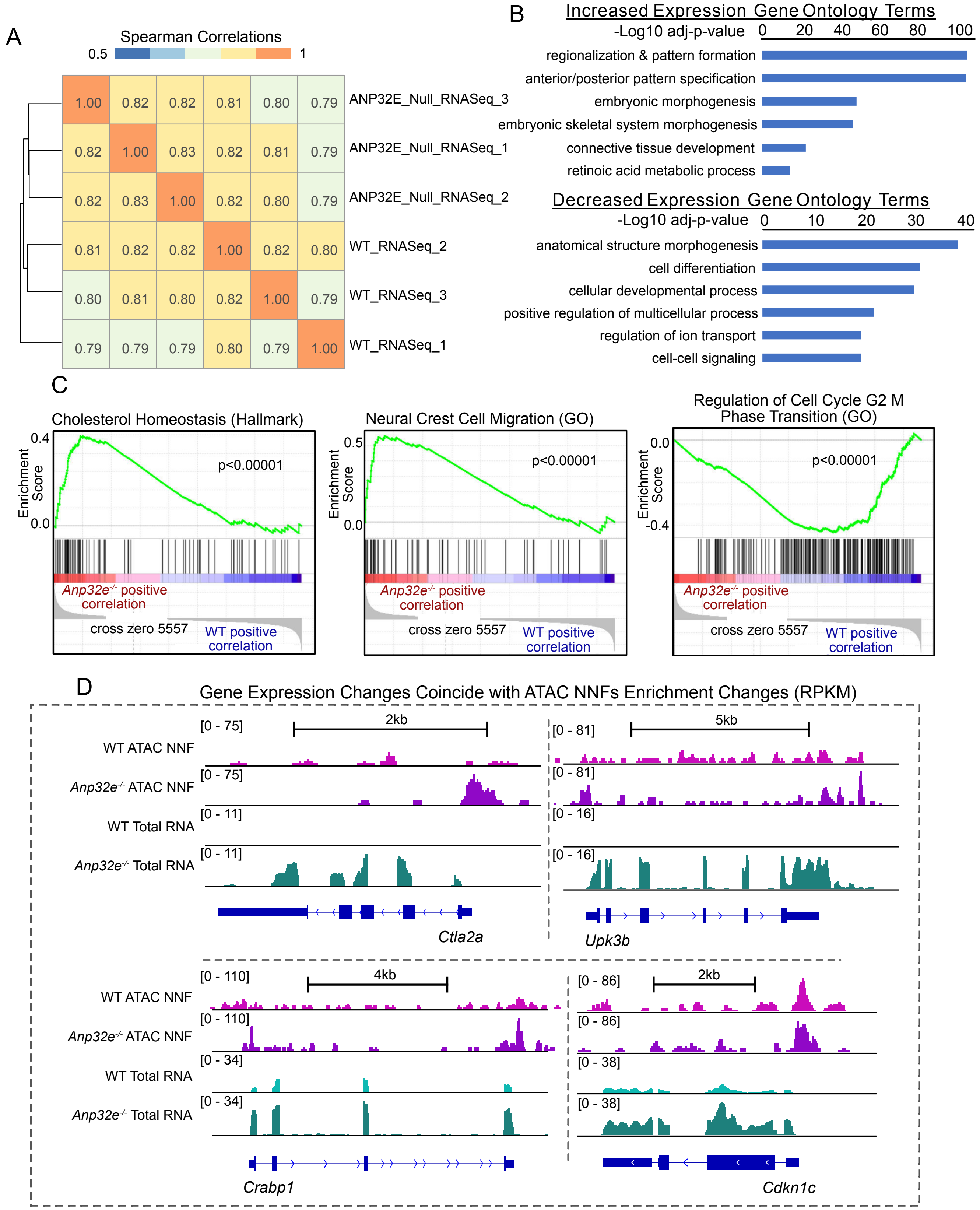

ANP32E_Null_RNASeq_3	1.00	0.82	0.82	0.81	0.80	0.79
ANP32E_Null_RNASeq_1	0.82	1.00	0.83	0.82	0.81	0.79
ANP32E_Null_RNASeq_2	0.82	0.83	1.00	0.82	0.80	0.79
WT_RNASeq_2	0.81	0.82	0.82	1.00	0.82	0.80
WT_RNASeq_3	0.80	0.81	0.80	0.82	1.00	0.79
WT_RNASeq_1	0.79	0.79	0.79	0.80	0.79	1.00

Supplemental Figure 7. Loss of ANP32E causes transcriptional dysregulation.

(A) Heatmap of pairwise Spearman correlation values showing high correlation among RNA sequencing replicates.
(B) Enriched gene ontology terms for increased and decreased transcripts in *Anp32e*$^{-/-}$ MEFs.
(C) Gene set enrichment analysis of *Anp32e*$^{-/-}$ compared to WT MEF RNA expression data revealed several gene sets and examples are shown. (Nominal p-values were generated based on statistical significance of individual gene sets without correction)
(D) Snapshots of genome browser views of non-nucleosome fragment (NNF) of chromatin accessibility and RNA-Seq of both WT and *Anp32e*$^{-/-}$ MEFs.

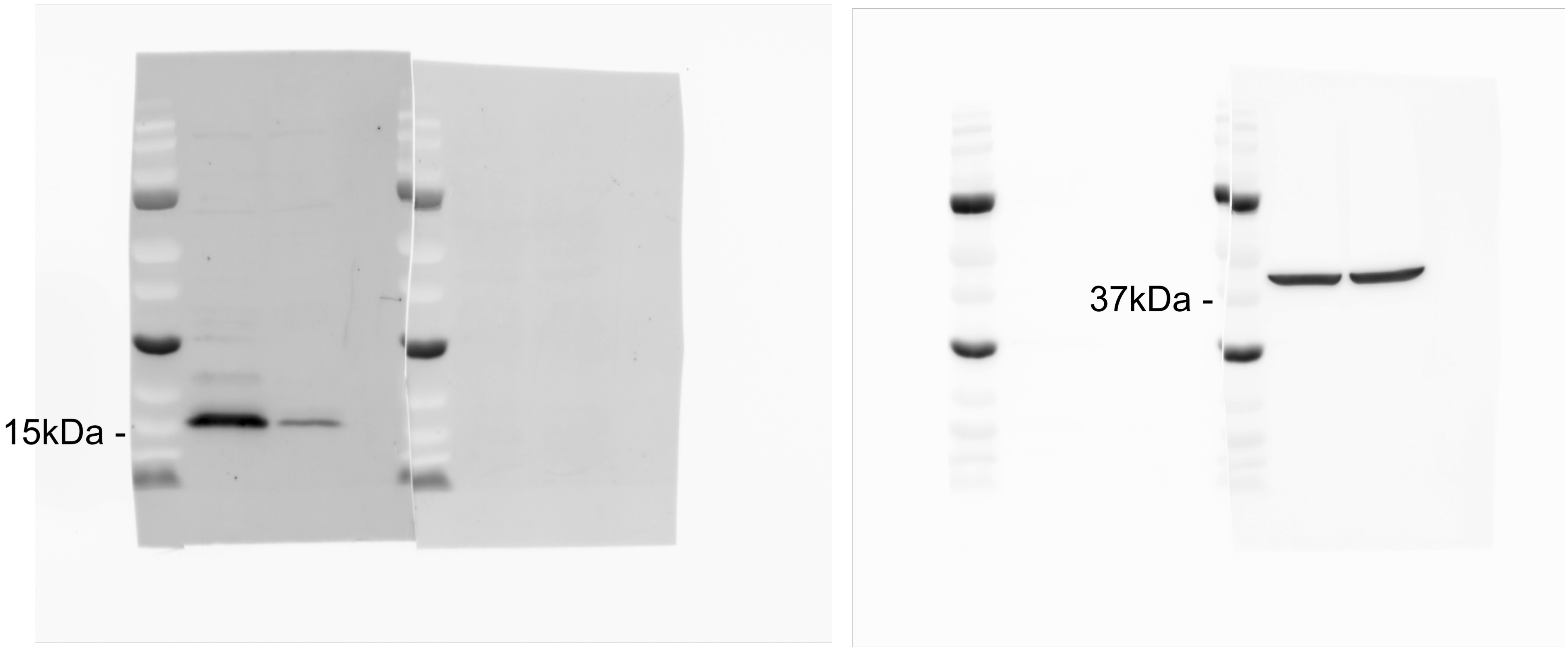

Supplementary Figure 8. Original Scans from Western Blots corresponding to Figure 4A.

Original Western blots are displayed for H2A.Z (left) and beta-Actin (right). In each blot the lane immediately to right of the molecular weight marker is control siRNA knockdown and the next lane is H2A.Z siRNA knockdown.

Supplementary Table 1. Dataset availability.

Dataset	Cell type	Source	Identifier	Link
ATAC-Seq of wt and Anp32e null MEF	MEF	This study	GEO: GSE145705	https://www.ncbi.nlm.nih.gov/geo/query/acc.cgi?acc=GSE145705
RNA-Seq of wt and Anp32e null MEF	MEF	This study	GEO: GSE145705	https://www.ncbi.nlm.nih.gov/geo/query/acc.cgi?acc=GSE145705
ATAC-Seq of non-targeting and H2A.Z siRNA knockdown Anp32e null MEF	MEF	This study	GEO: GSE145705	https://www.ncbi.nlm.nih.gov/geo/query/acc.cgi?acc=GSE145705
SP1 CUT&Tag of wt and Anp32e null MEF	MEF	This study	GEO: GSE145705	https://www.ncbi.nlm.nih.gov/geo/query/acc.cgi?acc=GSE145705
SMARCA4 CUT&Tag of wt and Anp32e null MEF	MEF	This study	GEO: GSE145705	https://www.ncbi.nlm.nih.gov/geo/query/acc.cgi?acc=GSE145705
ANP32E CUT&Tag of wt MEF	MEF	This study	GEO: GSE145705	https://www.ncbi.nlm.nih.gov/geo/query/acc.cgi?acc=GSE145705
H2A.Z ChIP-Seq of wt and Anp32e null MEF	MEF	Obri A, et al, 2014	GEO: GSE51579	https://www.ncbi.nlm.nih.gov/geo/query/acc.cgi?acc=GSE51579
H3K27me3 ChIP-Seq	MEF	Xie W, et al, 2015	GEO: GSE72237	https://www.ncbi.nlm.nih.gov/geo/query/acc.cgi?acc=GSE72237
H3K27Ac ChIP-Seq	MEF	Xie W, et al, 2015	GEO: GSE72237	https://www.ncbi.nlm.nih.gov/geo/query/acc.cgi?acc=GSE72237
H3K4me3 ChIP-seq	MEF	Xie W, et al, 2015	GEO: GSE72237	https://www.ncbi.nlm.nih.gov/geo/query/acc.cgi?acc=GSE72237
SMARCA4 ChIP-Seq	MEF	Vierbuchen T, et al, 2017	GEO: GSM2905665	https://www.ncbi.nlm.nih.gov/geo/query/acc.cgi?acc=GSM2905665
H2A.Z ChIP-Seq	mESC	Hsc CC, et al, 2018	GEO: GSE100460	https://www.ncbi.nlm.nih.gov/geo/query/acc.cgi?acc=GSE100460
ATAC-Seq	mESC	Juric I, et al, 2019	GEO: GSE119663	https://www.ncbi.nlm.nih.gov/geo/query/acc.cgi?acc=GSE119663
RNA-Seq	mESC	Hsc CC, et al, 2018	GEO: GSE100460	https://www.ncbi.nlm.nih.gov/geo/query/acc.cgi?acc=GSE100460
H3K27me3 ChIP-Seq	mESC	Hsc CC, et al, 2018	GEO: GSE100460	https://www.ncbi.nlm.nih.gov/geo/query/acc.cgi?acc=GSE100460
H3K27Ac ChIP-Seq	mESC	Hsc CC, et al, 2018	GEO: GSE100460	https://www.ncbi.nlm.nih.gov/geo/query/acc.cgi?acc=GSE100460
H3K4me3 ChIP-seq	mESC	Hsc CC, et al, 2018	GEO: GSE100460	https://www.ncbi.nlm.nih.gov/geo/query/acc.cgi?acc=GSE100460
