# Supplementary material for: Genome-wide chromatin accessibility is restricted by ANP32E
Source: Nat Commun. 2020 Oct 8;11:5063. doi: 10.1038/s41467-020-18821-x (PMC7546623; doi:10.1038/s41467-020-18821-x)
Supplement: Supplementary file 3 — Reporting Summary [file 41467_2020_18821_MOESM3_ESM.pdf]

nature research

Corresponding author(s): Patrick J Murphy

Last updated by author(s): Sep 7, 2020

Reporting Summary

Nature Research wishes to improve the reproducibility of the work that we publish. This form provides structure for consistency and transparency in reporting. For further information on Nature Research policies, see our Editorial Policies and the Editorial Policy Checklist.

Statistics

For all statistical analyses, confirm that the following items are present in the figure legend, table legend, main text, or Methods section.

n/a	Confirmed	
☐	☒	The exact sample size (*n*) for each experimental group/condition, given as a discrete number and unit of measurement
☐	☒	A statement on whether measurements were taken from distinct samples or whether the same sample was measured repeatedly
☐	☒	The statistical test(s) used AND whether they are one- or two-sided<br>*Only common tests should be described solely by name; describe more complex techniques in the Methods section.*
☐	☒	A description of all covariates tested
☐	☒	A description of any assumptions or corrections, such as tests of normality and adjustment for multiple comparisons
☐	☒	A full description of the statistical parameters including central tendency (e.g. means) or other basic estimates (e.g. regression coefficient) AND variation (e.g. standard deviation) or associated estimates of uncertainty (e.g. confidence intervals)
☐	☒	For null hypothesis testing, the test statistic (e.g. *F*, *t*, *r*) with confidence intervals, effect sizes, degrees of freedom and *P* value noted<br>*Give P values as exact values whenever suitable.*
☒	☐	For Bayesian analysis, information on the choice of priors and Markov chain Monte Carlo settings
☒	☐	For hierarchical and complex designs, identification of the appropriate level for tests and full reporting of outcomes
☒	☐	Estimates of effect sizes (e.g. Cohen's *d*, Pearson's *r*), indicating how they were calculated

Our web collection on statistics for biologists contains articles on many of the points above.

Software and code

Policy information about availability of computer code

Data collection	Data were collected using high-throughput sequencing from NextSeq, NovaSeq, and HiSeq platforms, with bcl2fastq (V2.20) software available from Illumina.
Data analysis	Sequencing data was analyzed with commonly used open source software methods. These include: Bowtie2 – v2.3.4.1, Picard – v2.18.23, Deeptools – v3.1.3, Bedtools - v2.29.2, MACS2 – v2.1.4, DiffBind - v2.14.0, IGV – v2.4.16, R – v3.6.1, Hint-ATAC - v0.12.3, nucleoATAC - v0.3.4, ucsc-binary-utilities – v373, GREAT – v4.0.4, PANTHER - v14.0, DESeq2 - v1.26.0, GSEA – v4.0.1, RNA-STAR – v2.7.3a, eulerr - v6.1.0, pheatmap - v1.0.12

For manuscripts utilizing custom algorithms or software that are central to the research but not yet described in published literature, software must be made available to editors and reviewers. We strongly encourage code deposition in a community repository (e.g. GitHub). See the Nature Research guidelines for submitting code & software for further information.

Data

Policy information about availability of data

All manuscripts must include a data availability statement. This statement should provide the following information, where applicable:

- Accession codes, unique identifiers, or web links for publicly available datasets
- A list of figures that have associated raw data
- A description of any restrictions on data availability

Sequencing data is publicly available through NIH GEO Datasets: GSE145705

Field-specific reporting

Please select the one below that is the best fit for your research. If you are not sure, read the appropriate sections before making your selection.

- [x] Life sciences
- [ ] Behavioural & social sciences
- [ ] Ecological, evolutionary & environmental sciences

For a reference copy of the document with all sections, see nature.com/documents/nr-reporting-summary-flat.pdf

Life sciences study design

All studies must disclose on these points even when the disclosure is negative.

Sample size	To ensure robust measurements of variation, and to facilitate statistical comparisons between samples of different types, 3 biological replicates were used for cell culture studies.
Data exclusions	More than 36 high throughput sequencing datasets were generated in this study, and only one dataset was excluded. From initial analysis of the H2A.Z knockdown ATAC-Seq datasets, we determined that one of our biological replicates was over-tagmented. This sample was therefore compromised, and further inclusion of this dataset in our study would have introduced artifacts and inhibited our analyses.
Replication	Cell culture methods were used such that each experiment was done using a minimum of 3 biological replicates. When possible, orthogonal experimental methods were used in an attempt to replicate results. All attempts at replication were successful.
Randomization	All statistical comparisons were made between test datasets and their appropriate negative controls or background unparsed datasets. For this reason, no randomization was necessary.
Blinding	Samples were blinded prior to submitting for high throughput sequencing and during the initial alignment stages. Subsequently, bioinformatics measurements took place in a controlled unbiased and unsupervised manner.

Reporting for specific materials, systems and methods

We require information from authors about some types of materials, experimental systems and methods used in many studies. Here, indicate whether each material, system or method listed is relevant to your study. If you are not sure if a list item applies to your research, read the appropriate section before selecting a response.

Materials & experimental systems

n/a	Involved in the study	
[ ]	[x]	Antibodies
[ ]	[x]	Eukaryotic cell lines
[x]	[ ]	Palaeontology and archaeology
[x]	[ ]	Animals and other organisms
[x]	[ ]	Human research participants
[x]	[ ]	Clinical data
[x]	[ ]	Dual use research of concern

Methods

n/a	Involved in the study	
[x]	[ ]	ChIP-seq
[x]	[ ]	Flow cytometry
[x]	[ ]	MRI-based neuroimaging

Antibodies

Antibodies used	H2A.Z antibody - Active Motif 39113, beta-Actin antibody - Sigma A5441 clone AC-15, ANP32E antibody - Thermo PA542860, SMARCA4 antibody - Abcam ab110641, SP1 antibody - Santa Cruz sc-17824 clone E-3
Validation	Antibodies were validated in the following prior publications: H2A.Z Shin et al. 2018 DOI: 10.1016/j.bbagrm.2018.03.002, beta-Actin Rugg-Gunn et al. 2010: DOI: 10.1073/pnas.0914507107, SMARCA4: Friman et al. 2019 DOI: 10.7554/eLife.50087, SP1 Calabrese et al. 2012 DOI: 10.1016/j.cell.2012.10.037. For ANP32E we preformed western blotting assays to confirm specificity prior to ChIP studies.

Eukaryotic cell lines

Policy information about cell lines

Cell line source(s)	Primary mouse embryonic fibroblasts were from WT and Anp32e-/- embryos and graciously donated to us by Dr. Ali Hamiche, at the Institut de Génétique et de Biologie Moléculaire et Cellulaire, Cedex, France
Authentication	Using RNA-Seq we validated the Anp32e mutation originally published Reilly et al. 2010.
Mycoplasma contamination	None

Commonly misidentified lines (See ICLAC register)	NA
