# Supplementary material for: Genome-wide chromatin accessibility is restricted by ANP32E
Source: Nat Commun. 2020 Oct 8;11:5063. doi: 10.1038/s41467-020-18821-x (PMC7546623; doi:10.1038/s41467-020-18821-x)
Supplement: Supplementary file 2 — Peer Review File [file 41467_2020_18821_MOESM2_ESM.pdf]

Reviewers' comments:

Reviewer #1 (Remarks to the Author):

This manuscript from Murphy et al addresses the question of H2AZ restriction by ANP32E. Their analyses confirm previous reports that ANP32E restricts H2AZ accumulation. The main contribution is the finding that enrichment of H2AZ at the -1 nucleosome appears to be preferentially restricted by ANP32E. Overall, this submission provides mainly computational assessment of existing H2AZ ChIP data sets and newly acquired ATAC-seq and RNA-seq data sets from ANP32E-/- MEFs. While the analyses seem sound, the effects are quite subtle, and the submission suffers from lack of experimental validation or explanation of key findings, specifically ANP32E's preference for preventing H2AZ accumulation at the -1 nucleosome and validation of enriched TF binding based on subnucleosomal ATAC-seq analysis.

Major Conclusions and Comments:
1. The first point that the authors make is that H2AZ and ATAC mostly overlap in MEF (with a slightly different interpretation in ESC). The authors make this point because there are several reports in the literature that H2AZ, although generally associated with gene activation, has also been associated with increased nucleosome stability and polycomb-mediated gene silencing in ESCs. Generally, these data seem sound, and I think that this is an important analysis. Related, do the authors have access to RNA-seq from ANP32E-/- ESCs and see any transcriptional effect at bivalent promoters? In the last two panels of Figure 1, the authors make the point that only overlapping H2AZ and ATAC peaks are associated with high levels of transcription. Can the authors provide RPKM for each data set at each set of promoters? My prediction is that the non-overlapping peaks have relatively low signal.

2. The authors then begin analysis of the effects of ANP32E on chromatin accessibility. First, the loss of ANP32E in MEF is not validated in this submission - RNA-seq or WB should be sufficient. Here, in addition to loss of ANP32E leading to many regions with more accessible chromatin, there are a substantial number of peaks that become "closed" in ANP32E-/- (~25% of the "opened" regions). Can the authors comment? Also, it seems that most open regions become more open by ATAC-seq without really changing H2AZ enrichment. The authors show a small increase in H2AZ enrichment in Q4, but are the 47K regions that become more open show statistical increase in H2AZ? Further, in Fig. S2C/D, all regions show increased accessibility regardless of what happens to H2AZ in the APN32E-/-. This is discussed by the authors, but never explained, besides invoking "additional factors" as being responsible.

3. Figure 3 is really the main finding of the submission, that H2AZ accumulates at the -1 nucleosome in ANP32E-/- MEFs. From Fig. 3A, this result looks really quite modest (not more than a 6% change). Further, it's not clear what the beds are that are used for Fig. 3B, how many regions fall into each category, and the scale (normalized frequency doesn't seem terribly meaningful). The major sticking point is that it is never addressed why ANP32E should prefer to prune/restrict the -1 nucleosome, which is what I feel the authors would need to provide for this to be a significant advance. The authors say they favor a mechanism that ANP32E prevents deposition, but they do not provide evidence to support that model, which they depict in their final figure.

4. It'd be nice to have some experimental validation of the SMARCA4 result – does nucleosome positioning change with SMARCA4 KD? How about SMARCA4 KD in ANP32E-/-? And SMARCA4 enrichment in the ANP32E-/-? Also, if the model is that SMARCA4 is more likely to be bound at an H2AZ nucleosome, what about the relatively inverted binding pattern observed for enhancers and promoters for H2AZ and SMARCA4?

5. The authors perform analyses of subnucleosomal fragments from ATAC-seq to show that a number of motifs become less protected in ANP32E-/- MEF. It would be really nice to see validation of this prediction with ChIP of any of these TFs in the ANP32E-/- MEF. Related, in the abstract, the authors

claim that they observe "heightened transcription factor binding", which is not in reality what the ATAC-seq assay is measuring.

Minor points:
1. I found MESC confusing. This cell type is typically referred to in the literature as ESC, and the authors could use mESC if they want to specifiy species.
2. It would be helpful to have a table listing all of the data sets used in the analysis and where they came from. It is not clear whether the authors are using the same cell lines for every ChIP-seq, ATAC-seq and RNA-seq data analyzed. Otherwise is hard to compare all of them.
3. The heatmaps in all the figures do not clearly state which regions they are representing. Throughout, the authors should make more clear the exact nature of the regions being processed for each figure. Beds should be provided as supplemental tables, sorry if I missed these and they were present.
4. Scales should be included for browser tracks.
5. Please clearly define enhancer and promoter definitions in the text.
6. Please check carefully the description of the figures in the text, there are multiple mistakes (bottom/top/left/right). Also, I noticed a number of grammatical typos throughout.
7. Please check carefully the figure legends, there are multiple mistakes (exp Fig. 1F is the same as panel E not D; Fig 6D).
8. It's not always clear whether authors are referring to mESCs or MEFs, and this can make interpreting the results quite confusing.
9. Throughout, I'm not sure how informative the ontology analysis is.

Reviewer #2 (Remarks to the Author):

ANP32E is an H2A.Z chaperone that is part of P400 complex. Murphy et al. examine genome-wide accessibility and expression changes upon loss of ANP32E and correlate these changes to changes in H2A.Z profiles to understand how H2A.Z deposition interplays with genomic accessibility and gene expression. They conclude that loss of ANP32E increases H2A.Z levels and positioning which could result in increased TF binding, and increased gene expression.
The effect of the presence of H2A.Z in a nucleosome on chromatin accessibility, gene activation, and transcription elongation is an important unresolved question with previous work discussing both an activating and inhibiting role of H2A.Z on chromatin accessibility. Thus, this study is timely by comparing H2A.Z dynamics, chromatin accessibility and gene expression in vivo. However, due to limitations in the data analyses presented, their conclusions are not fully supported. I discuss below to what extent the data/analyses they present support their main conclusions and how to improve the analyses to better support their conclusions:

1. H2A.Z resides at active genes and highly accessible chromatin regions.

The Authors show a positive correlation between chromatin accessibility as measured by ATAC-seq and H2A.Z occupancy as measured by ChIP-seq. The presented data supports their conclusion that H2A.Z levels correlate with accessibility in mESCs and MEFs.

2. Loss of ANP32E leads to a widespread increase in chromatin accessibility.
a. The Authors show clearly that there is a global increase in promoter accessibility upon loss of ANP32E, and genome-wide, the number of sites with higher accessibility is far greater than sites with decreased accessibility upon loss of ANP32E. For Figure 2B, volcano plot that incorporates p-values determined from fold changes across replicates would be better compared to showing presumably the average across the replicates. Furthermore, a 2D histogram should be shown to avoid overplotting as seen in scatterplot.

b. However, the correlation between H2A.Z changes and accessibility changes is weak, which is acknowledged by the Authors. All classes of H2A.Z changes result in increased accessibility, which point to the possibility that the majority of effects of ANP32E loss may be beyond changes in H2A.Z occupancy. An alternate explanation is the fact that there could be global change in H2A.Z levels upon loss of ANP32E which will be invisible in ChIP-seq. Immunoblot of chromatin associated H2A.Z in WT and ANP32E will show if there is global change in H2A.Z upon loss of ANP32E. Computationally, methods that normalize ChIP-seq signals based on background regions might uncover global changes. If this is the case, the correlation between changes in H2A.Z and changes in accessibility could be stronger when accounting for global change in H2A.Z compared to what is presented currently.

3. Positional hierarchy of H2A.Z at promoters coincides with differences in gene function.
a. Change in H2A.Z upon ANP32E loss is shown (Figure 3A). It is unclear why genes on forward strand and reverse strand are plotted separately instead of being combined and plotted in the direction of transcription. Secondly, the overall effect of ANP32E loss on H2A.Z is only a ~5-6% increase in the promoter - and it is not clear if this increase is statistically significant. This weak change in H2A.Z upon loss of ANP32E indicates that the dramatic effects on accessibility may be independent of changes in H2A.Z occupancy.
b. For Figure 3B, y-axis values are absent, so the extent of changes cannot be assessed, and no statistical analysis is presented to show that the differences are significant. Furthermore, it is not clear how "Normalize Frequency" is calculated. In Figure S2C, D, the range is 0 to 1 for all 3 plots - the exact steps of normalization should be described in the methods.
c. For Figure S3C, it is not clear how many promoters belong to each class in WT H2A.Z. Also, it is not clear if changes in proportions are statistically significant.
d. For Figure 3C, it is not clear if the p-values for GO enrichments are corrected for multiple testing.

It is difficult to assess if the analyses in Figure 3, S3 support the Author's conclusions due to missing information pointed above.

4. H2A.Z hierarchy changes correspond with accessibility and nucleosome positioning changes.

a. It is difficult to untangle increase in accessibility of +1 nucleosome versus increased positioning of +1 nucleosome with data as presented and described in Figure 4A (y-axis values missing here also). To check changes in positioning, distribution of width of ATAC peak at +1 nucleosome across genes in WT and ANP32E-/- might be more convincing.
b. The Authors show a correlation of SMARCA4 ChIP-seq and H2A.Z ChIP-seq in WT and propose "these results are consistent with a nucleosome remodeling mediated mechanism where H2A.Z promotes increased nucleosome positioning flanking the TSS to stimulate chromatin accessibility at the NDR". However, these results could also be consistent with loss of SMARCA4 activity in Anp32E-/- leading to increased positioning. Since only correlation in WT is presented, both possibilities should be discussed in the text.

5. TF-footprinting increases adjacent to better positioned H2A.Z nucleosomes.

a. Lack of y-axis values in Figure 5A prevent assessment of the magnitude of increase of NNFs due to ANP32E loss - also it should be determined if the increase in NNF is statistically significant using ATAC replicates. In Figure 5B, the change in NNF is shown only for changes from one class of promoters, it is not clear if other classes have expected changes in NNF. Even for the plot shown the effect is very weak and no statistics are shown.
b. For Figure 5C, it is not clear what the scale of differential protection scores is - what does a 0.05 differential protection score mean in terms of inferred TF binding? Without this information, it is difficult to assess the effect size. The effect sizes in 5D are extremely weak, indicating minor increases in inferred TF binding due to loss of ANP32E.
c. Finally, the title of this section indicates binding adjacent to better positioned H2A.Z nucleosomes. However, no analysis is presented comparing positioning of H2A.Z nucleosomes and TF binding as

inferred from ATAC-seq.

6. ANP32E loss leads to dysregulation of genes involved in differentiation.
a. "Consistent with H2A.Z functioning as an activator, gene promoters with increased accessibility tended to be up-regulated, and promoters with reduced accessibility tended to be down-regulated (Fig 6B)." The comparison shown in 6B is between accessibility and gene expression, hence it is not clear why this says anything about H2A.Z as an activator. Furthermore, this data doesn't rule out the possibility that gene activity sets H2A.Z levels. Hence, both possibilities should be discussed.
b. There are robust changes in expression (many genes with Log2FC>1 in Figure 6A), but median log2FC in top quintile of ATAC accessibility is barely above 0, indicating very low correlation of ATAC changes with RNA changes as presented.
c. In Figure 6D, it is not clear if GO enrichment p-values were corrected for multiple testing. Multiple testing corrections is essential for all GO analyses in the study.

Additional Comments:

1. For sake of reproducibility of the analyses, information on use of published data should be expanded beyond just citations. The exact GEO id for each external dataset used should be part of methods or as a Supplementary Table.
2. For all line plots and genomic snapshots, y axis needs to be present and methods should describe how the values on the y axes were arrived at.

Reviewer #3 (Remarks to the Author):

The manuscript by Murphy and Meng et al. presents a study of H2A.Z organization and chromatin accessibility through the perturbation of p400/TIP60 complex subunit ANP32E in mouse embryonic fibroblasts. They show that ANP32E loss greatly increases chromatin accessibility genome wide and that accessibility increases regardless of changes in H2A.Z enrichment. The authors categorized H2A.Z marked promoters based on relative changes in H2A.Z at the +1 and -1 nucleosome in response to ANP32E loss. They conclude that H2A.Z localization at the TSS obeys a positional hierarchy wherein initial deposition occurs at the +1 nucleosome (Class One) and accumulates at the -1 nucleosome only after the +1 nucleosome is "saturated" with H2A.Z (Class Two). They conclude this process occurs in an ANP32E dependent manner. The authors then perform GO analysis on transitions from Class One to Class Two promoters between WT and ANP32E null MEFs as well as between MESC and MEFs and find genes associated with developmental differentiation in both instances. They go on to show that ANP32E loss leads to increased nucleosome positioning at promoters where H2A.Z enrichment increases at the +1 or the -1 nucleosome suggesting that H2A.Z promotes chromatin accessibility through improved nucleosome positioning. The authors used non-nucleosomal chromatin fragments (NNFs) as a proxy for TF binding and found that NNFs increased in Anp32e -/- cells compared to WT and these NNFs were most abundant at Class Two promoters. Further TF-footprinting analysis revealed an increase in putative TF binding events after ANPE32E loss. RNA-seq analysis found ANP32E as a key regulator in developmental gene expression. Overall, the manuscript introduces insights into the role H2A.Z plays in chromatin state at promoters and introduces an interesting theory on ANP32E's role in H2A.Z-mediated regulation. With the following adjustments I think this manuscript will be fit for publication.

Major Points
i. Figure 4
a. "these results are consistent with a nucleosome remodeling mediated mechanism where H2A.Z promotes increased nucleosome positioning flanking the TSS to stimulate chromatin accessibility at the NDR."
i. Shown previously in Figure S2 B-D, accessibility increases regardless of changes in H2A.Z. This

figure needs a control analysis to show that loss (or no change) of H2A.Z does not also increase nucleosome positioning at promoters. Conclusions made here and downstream may need be reworked depending on the results of this control analysis.
ii. Figure 3D
a. “One interesting possibility is that ANP32E levels and H2A.Z positioning are important for defining cellular function during differentiation and potentially for regulating H2A.Z positioning during cellular transitions. Consistent with this possibility, Anp32e mRNA is considerably lower in MEFs compared with MESCs (Fig 3D), and H2A.Z is much more restricted to the +1 nucleosome position in MESCs compared with MEFs”
i. Postulations made using Figure 3D could be further validated by experiments involving overexpression of ANP32E in MEF and observing H2A.Z/accessibility changes at select candidate loci.
iii. Figure 3
a. Seems like there was some opportunity lost for some reciprocal validation here in Figure 3.
i. For instance, if you looked at promoters in the top 25% of +1 nucleosome H2A.Z enrichment in WT, you would expect to see H2A.Z spread to the -1 nucleosome at these same promoters in Anp32e-/-
iv. Figure 5
a. The authors showed that NNFs accumulated more at promoters that transitioned from Class One to Class Two in Anp32e-/-. They then identified several motifs with positive HINT scores, indicating increased TF binding. However, the authors fail to connect these putative changes in TF binding back to changes in H2A.Z enrichment and therefore fail to reinforce their conclusions on H2A.Z promoting nucleosome positioning established in the previous figure.
b. Additionally, this figure in particular would benefit from ChIP-qPCR experiments for candidate TFs to validate the proposed increased TF binding inferred by the HINT analysis.

Minor Points
v. Introduction
a. The first paragraph of the intro seems quite overwrought. For example, the statement that “whether particular factors regulate widespread chromatin state remains largely unknown” is simply not true. Likewise, for the sentence, “…removal of factors thought to function in regulation of chromatin accessibility, including H3K27ac, H3.3, and prominent nucleosome remodelers, had only modest impacts on genome-wide chromatin states”. Aside from the issue of defining what a “modest” impact is, the two references cited in this sentence could be counterbalanced by many studies of remodelers, polycomb group proteins, and histone variants like cenH3, that do indeed have widespread effects on chromatin. Perhaps a better use of space here is to simply introduce the general topic in a more even-handed way.
vi. Figure 1E
a. What defines an accessible/H2A.Z enriched promoter? Peak overlap? If so, by how much?
vii. Figure S1D
a. “These results indicate that H2A.Z is consistently correlated with chromatin accessibility, as well as with marks of gene activation H3K4me3 and H3K27ac, but H2A.Z is only associated with the gene silencing mark H3K27me3 in the context of bivalent chromatin, at a subset of locations in MESCs.”
i. I don’t think this claim can be appropriately made based on how the data are presented.
viii. Figure 2B-C
a. Reduced H2AZ and increased accessibility “represented the extreme minority of regions, and chromatin changes at these sites were much less significant than changes at all other sites”
i. First, this is almost a quarter of all peaks.
ii. Second, “much less significant” implies statistical analysis. Consider rephrasing.
ix. Figure 3A
a. Include “H2A.Z” in plot title to orient reader
b. Consider changing y-axis to fold change as opposed to percentage.

x. Figure S3A
a. Several analyses in the paper are divided into +/- strand transcription. This division should be justified in the text.

xi. Figure 3B
a. Fig 3B depicts C1 to C2 (top) and C0 to C1 (bottom) transitions. However, Fig 3C depicts GO analysis for C1->C2 and C1->C0 transitions. Displayed in this way, the reader has no insight into what a 1->0 transition looks like from WT to Anp32e-/- cells.
b. Here and many other places, the y-axis is labeled 'normalize frequency", but this should be "normalized frequency"
xii. Fig 6B
a. "Consistent with H2A.Z functioning as an activator, gene promoters with increased accessibility tended to be up-regulated, and promoters with reduced accessibility tended to be down-regulated"
i. How exactly is that consistent with H2AZ's function as an activator? Analysis in Figure 2 showed positive or negative changes in H2A.Z lead to increased accessibility.
xiii. Fig 6C
a. "Furthermore, we observed a more dramatic gene expression change when we restricted our analysis to genes in closest proximity to intergenic motifs identified by footprinting analysis (Fig 6C), indicating that the ANP32E mediated changes at intergenic regulatory regions, such as enhancers, may be a major contributor to the gene expression changes we observed."
i. Axis should be consistent between B and C to avoid misleading the reader.
xiv. Discussion
a. "Class Zero promoters lack H2A.Z, have poorly positioned nucleosomes, and are largely inaccessible."
i. Class Zero does lack H2AZ but nucleosome position was never analyzed at this class.
b. "Furthermore, H2A.Z marked gene promoters that gained more H2A.Z in the absence of ANP32E, and became more accessibility, tended to be up-regulated"
i. Typo: accessibility -> accessible
c. Claims in discussion should cite relevant Figures
xv. Method
a. "Promoters with increased H2A.Z at +1 nucleosome are defined as log2FC values in top 25% at -1 nucleosome and bottom 75% at +1 nucleosome."
i. Typo: should say "top 25% at +1 nucleosomes and bottom 75% at -1 nucleosomes."
b. "Data were compared with data from studies which utilized Chromatin Immuno-Precipitation Sequencing (ChIP-Seq), but no new ChIP-Seq datasets were generated from this study. Data from numerous published studies were utilized 24,32,64–66."
i. Each dataset used should be more explicitly stated in text.

We would like to begin by thanking the editors and reviewers for their efforts in helping us to publish this study. We found the reviewers to be thoughtful, fair, and critical. We found each of the reviewer's comments and concerns to be very useful in guiding us through the revision process. We have now accommodated all reviewers' concerns, and we are confident that our manuscript has been significantly improved as a result. Below we have addressed each issue point-by-point, and our specific responses can be found beneath each noted concern highlighted in blue text.

Reviewer 1
Main Concerns:

1. The first point that the authors make is that H2AZ and ATAC mostly overlap in MEF (with a slightly different interpretation in ESC). The authors make this point because there are several reports in the literature that H2AZ, although generally associated with gene activation, has also been associated with increased nucleosome stability and polycomb-mediated gene silencing in ESCs. Generally, these data seem sound, and I think that this is an important analysis. Related, do the authors have access to RNA-seq from ANP32E-/- ESCs and see any transcriptional effect at bivalent promoters? In the last two panels of Figure 1, the authors make the point that only overlapping H2AZ and ATAC peaks are associated with high levels of transcription. Can the authors provide RPKM for each data set at each set of promoters? My prediction is that the non-overlapping peaks have relatively low signal.

> We thank Reviewer 1 for affirming the importance of our H2A.Z measurements in mESCs vs. MEFs. We agree that it was important for us to assess how H2A.Z might function differently in stem cells compared with differentiated cells. Unfortunately, we do not have access to *Anp32e*-/- mESCs or an RNA-Seq dataset from these cells. As the reviewer suggests, it will be important for future studies to investigate differences in H2A.Z function in different cell types or during developmental progression.
>
> We would especially like to thank Reviewer 1 for their suggestion to measure RPKM in overlapping and non-overlapping H2A.Z and ATAC peaks. This was an excellent suggestion. We have now done this analysis, and as Reviewer 1 predicted, the non-overlapping peaks have relatively low signal (Figure 1F & 1G). This result further supports the strong association between H2A.Z enrichment and chromatin accessibility.

2. The authors then begin analysis of the effects of ANP32E on chromatin accessibility. First, the loss of ANP32E in MEF is not validated in this submission - RNA-seq or WB should be sufficient. Here, in addition to loss of ANP32E leading to many regions with more accessible chromatin, there are a substantial number of peaks that become "closed" in ANP32E-/- (~25% of the "opened" regions). Can the authors comment? Also, it seems that most open regions become more open by ATAC-seq without really changing H2AZ enrichment. The authors show a small increase in H2AZ enrichment in Q4, but are the 47K regions that become more open show statistical increase in H2AZ? Further, in Fig. S2C/D, all regions show increased accessibility regardless of what happens to H2AZ in the APN32E-/-. This is discussed by the authors, but never explained, besides invoking "additional factors" as being responsible.

First, we would like to thank Reviewer 1 for noting a critical omission in our prior manuscript. In the acknowledgements we noted that the *Anp32e* null cells were generated from the null mouse and gifted to us by Dr. Ali Hamiche, but we failed to reference Reilly et al. *Plos One* 2010. We have now added this reference information to the main text, and for the sake of clarity, we have included an additional figure panel in Figure S2, as the reviewer suggests. This panel depicts the nature of the mutation, the reduction in *Anp32e* expression levels, and the absence of mRNA sequence corresponding to exons 2-5 – the H2A.Z interacting regions of ANP32E.

Second, the reviewer recommends we use an alternative statistical approach to assign differentially accessible regions in our analysis. Here again the reviewer makes an excellent suggestion. We have now used DiffBind for statistically assigning differential accessibility. DiffBind is similar to DeSeq2, but more appropriate for enrichment-based datasets. Using this more rigorous approach, we have now found that there are only 846 regions where accessibility decreased, and 18280 regions where accessibility increased in ANP32E deficient cells. Increases represent more than 95% of differentially accessible regions, further demonstrating the dramatic increase in accessibility we observed in ANP32E deficient cells. As a result of this improved analysis, there are no longer a substantial number of peaks that become "closed" in *Anp32e*-/- cells.

For the third point made by the reviewer, we agree that the H2A.Z enrichment changes we observed were much less dramatic than the observed chromatin accessibility changes. This important result was noted in the prior version of our manuscript, both in the 4th paragraph of the results section, and the end of the 3rd paragraph of the discussion. This result provided the rationale for our investigation of H2A.Z positioning at promoters (previous Figure 3). In the new version of our manuscript we investigate this result further both through additional analysis and new experimentation. We now provide measurements of accessibility changes as a distribution across all H2A.Z peaks (new Figure 2D), and find that accessibility does not increase at peaks where H2A.Z is lost in *Anp32e-/-*. In the new manuscript we also preformed additional experimentation, and demonstrate that H2A.Z depletion is able to rescue the majority of accessibility changes found at promoters (Figure 4 & S4). Taken together, these results indicate that ANP32E restricts chromatin accessibility in an H2A.Z dependent manner, even with only modest changes in H2A.Z enrichment.

3. Figure 3 is really the main finding of the submission, that H2AZ accumulates at the -1 nucleosome in ANP32E-/- MEFs. From Fig. 3A, this result looks really quite modest (not more than a 6% change). Further, it's not clear what the beds are that are used for Fig. 3B, how many regions fall into each category, and the scale (normalized frequency doesn't seem terribly meaningful). The major sticking point is that it is never addressed why ANP32E should prefer to prune/restrict the -1 nucleosome, which is what I feel the authors would need to provide for this to be a significant advance. The authors say they favor a mechanism that ANP32E prevents deposition, but they do not provide evidence to support that model, which they depict in their final figure.

We thank Reviewer 1 for noting the importance of our finding that H2A.Z changes occur at the -1 nucleosome position. When H2A.Z changes were measured across all promoters, even non-H2A.Z marked promoters, the aggregate level of change was shown to be quite modest, as noted by Reviewer 1. In the new version of the

manuscript we investigate this issue directly. In doing so, we performed new analysis methods (as suggested by Reviewer 3) and find that H2A.Z increases dramatically at the -1 nucleosome for regions with moderate H2A.Z levels in WT cells (the 60-80th percentile). H2A.Z increased by 25% (Log10FC = 0.1) at these regions. These results are perfectly aligned with our proposed H2A.Z hierarchy, and can be found in Figure S3A. In addition, we have more accurately labeled our figures and provided better descriptions of our bed files, as Reviewer 1 suggests. This new labeling strategy makes it clear in figure 3B, that aggregate RPKM increases more than 40% (from 9 to 13 RPKM enrichment) at the -1 nucleosome for promoters where H2A.Z is already present at the +1 nucleosome.

Additionally, we have revised the discussion section to make it clear that we do not intend to propose a model in which ANP32E prefers to prune/restrict the -1 nucleosome. Such a model would require that ANP32E engages in site specific H2A.Z eviction. Here ANP32E would either need to be targeted to the -1 nucleosome, or occur in high enough concentrations at very specific genomic sites to cause H2A.Z eviction specifically at the -1 nucleosomes. To date, no such targeting or site-specific eviction mechanisms have been described *in vivo*. Therefore, our model does not rely on ANP32E targeting, and a simpler well supported sequestration model seems more appropriate. In the discussion section we provide a better description for why we favor this model. Preferential installation of H2A.Z at the +1 nucleosome is well supported by published studies, for which we now include additional references. To further address the concern of Reviewer 1, we make note in the discussion that an eviction model remains a possibility, and importantly, the major conclusions from our study are not dependent on ANP32E functioning in H2A.Z eviction. We have updated our final model figure to summarize the major findings of the current study, which support ANP32E function in 1) restricting H2A.Z, 2) contributing to hierarchical localization of H2A.Z around the TSS, 3) inhibiting nucleosome positioning, and 4) restricting chromatin accessibility at the TSS of promoters.

4. It'd be nice to have some experimental validation of the SMARCA4 result – does nucleosome positioning change with SMARCA4 KD? How about SMARCA4 KD in ANP32E-/-? And SMARCA4 enrichment in the ANP32E-/-? Also, if the model is that SMARCA4 is more likely to be bound at an H2AZ nucleosome, what about the relatively inverted binding pattern observed for enhancers and promoters for H2AZ and SMARCA4?

We thank Reviewer 1 for this suggestion. Indeed, experimental validation of the SMARCA4 result was lacking in our prior manuscript, and in the revised version we have reconciled this issue. Studies in yeast indicate that several different nucleosome remodelers can be influenced by H2A.Z, including SWI/SNF and ISWI family members (Goldman 2010, Cakiroglu 2019). Additionally, studies have found that SMARCA2 is able to compensate for SMARCA4 loss (Wilson 2014). Based on these findings, rather than employing a loss-of-function approach, we used CUT&Tag to investigate how SMARCA4 enrichment changed in response to ANP32E loss, as suggested by Reviewer 1. In agreement with our hypothesis, we found there to be an overall increase in SMARCA4 enrichment at promoters upon ANP32E loss. These increases occurred at regions where accessibility increased and SMARCA4 enrichment decreased where accessibility decreased. These results can be found in Figure 6 and S6.

For the second point, we thank Reviewer 1 for noting this detail. Indeed, SMARCA4 enrichment is strongest at the NDR, while H2A.Z is enriched at the +1 and -1 nucleosome positions. This result was expected, as H2A.Z enrichment occurs in the context of nucleosomes, while SMARCA4 has been shown in several prior studies to be enriched at the TSS, where nucleosomes are lacking. Interestingly, in our study we find that SMARCA4 is highly enriched at H2A.Z marked promoters, and lacking at H2A.Z depleted promoters. In the revised version of this manuscript we provide additional measurements of this correlation, which can be found in figure S6A. Importantly, from our data we cannot distinguish between H2A.Z promoting SMARCA4 recruitment and H2A.Z simulating increased SMARCA4 remodeling, ultimately leading to additional SMARCA4 enrichment. We have therefore simplified our model figure such that it more accurately represents the results of our study (Figure 7E).

5. The authors perform analyses of subnucleosomal fragments from ATAC-seq to show that a number of motifs become less protected in ANP32E-/- MEF. It would be really nice to see validation of this prediction with ChIP of any of these TFs in the ANP32E-/- MEF. Related, in the abstract, the authors claim that they observe “heightened transcription factor binding”, which is not in reality what the ATAC-seq assay is measuring.

We thank Reviewer 1 for this suggestion. In the revised version of the manuscript we have validated that SP1 binding increases in the ANP32E lacking cells. SP1 binding increased at the same genomic SP1 motifs where accessibility increased. These results can be found in Figure 5B, S5A, and S5B. While Reviewer 1 is correct in their statement that ATAC-Seq studies do not measure TF binding, published studies using HINT-ATAC have indeed demonstrated that this technique is comparable to established DNase hypersensitivity methods for measuring widespread changes in TF binding (Li et al. *Genome Research* 2019). These studies in addition to our SP1 validations provide justification for our conclusion that TF binding is impacted by ANP32E loss.

<u>Minor Concerns:</u>

1. I found MESC confusing. This cell type is typically referred to in the literature as ESC, and the authors could use mESC if they want to specifiy species.

We agree that this abbreviation is not typical and could be confusing, so we have changed all instances of MESC to be mESC.

2. It would be helpful to have a table listing all of the data sets used in the analysis and where they came from. It is not clear whether the authors are using the same cell lines for every ChIP-seq, ATAC-seq and RNA-seq data analyzed. Otherwise is hard to compare all of them.

We thank Reviewer 1 for the suggestion and we have now added this table as a supplemental file (Table S1).

3. The heatmaps in all the figures do not clearly state which regions they are representing. Throughout, the authors should make more clear the exact nature of the

regions being processed for each figure. Beds should be provided as supplemental tables, sorry if I missed these and they were present.

We thank Reviewer 1 for the suggestion. We have modified our manuscript such that heatmaps now include the number of regions analyzed, and we have adjusted labeling to clarify the nature of regions. Throughout this study numerous datasets were parsed several times to test particular hypotheses and to perform numerous intersections. As a result, more than 100 unique bed files were generated with thousands of regions per bed file. Unfortunately, adding these bed files as a supplemental table is not practical nor is it typical for these types of studies. To better accommodate the reviewers request, however, we have updated the methods section to indicate how particular regions were parsed for analyses.

4. Scales should be included for browser tracks.

We have corrected this error and scales are now included for all browser tracks.

5. Please clearly define enhancer and promoter definitions in the text.

We modified text to include a definition for putative enhancers – all H3K27ac-occupied regions more than 2kb away from promoters in either mESCs or MEFs, as well as a definition for promoters – the region surrounding TSS +/- 1kb.

6. Please check carefully the description of the figures in the text, there are multiple mistakes (bottom/top/left/right). Also, I noticed a number of grammatical typos throughout.

We apologize for these mistakes. In the revised manuscript we have carefully checked for errors and have made our best attempt to correct typos throughout.

7. Please check carefully the figure legends, there are multiple mistakes (exp Fig. 1F is the same as panel E not D; Figure 6D).

We checked figure legends and corrected mistakes.

8. It’s not always clear whether authors are referring to mESCs or MEFs, and this can make interpreting the results quite confusing.

We have added explicit references to cell type in the main text.

9. Throughout, I’m not sure how informative the ontology analysis is.

We thank Reviewer 1 for this comment. We agree that gene ontology is not the most informative analysis for several of our figures. We moved ontology results to supplemental figures and de-emphasized these results, as suggested.

Reviewer 2

<u>Main Concerns:</u>

Loss of ANP32E leads to a widespread increase in chromatin accessibility.

a. The Authors show clearly that there is a global increase in promoter accessibility upon

loss of ANP32E, and genome-wide, the number of sites with higher accessibility is far greater than sites with decreased accessibility upon loss of ANP32E. For Figure 2B, volcano plot that incorporates p-values determined from fold changes across replicates would be better compared to showing presumably the average across the replicates. Furthermore, a 2D histogram should be shown to avoid overplotting as seen in scatterplot.

> We thank Reviewer 2 for this excellent suggestion. To accommodate this request, we have adjusted how we define differential accessibility, and we now provide a 2D volcano plot for Figure 2B, as requested. With this new analysis methods (using DiffBind) more than 95% of accessibility changes are increased. In addition to this new plot, we also provide heatmaps for accessibility measurements at differential regions in each biological replicate, showing in Figure S2C.

b. However, the correlation between H2A.Z changes and accessibility changes is weak, which is acknowledged by the Authors. All classes of H2A.Z changes result in increased accessibility, which point to the possibility that the majority of effects of ANP32E loss may be beyond changes in H2A.Z occupancy. An alternate explanation is the fact that there could be global change in H2A.Z levels upon loss of ANP32E which will be invisible in ChIP-seq. Immunoblot of chromatin associated H2A.Z in WT and ANP32E will show if there is global change in H2A.Z upon loss of ANP32E. Computationally, methods that normalize ChIP-seq signals based on background regions might uncover global changes. If this is the case, the correlation between changes in H2A.Z and changes in accessibility could be stronger when accounting for global change in H2A.Z compared to what is presented currently.

> We agree that changes in H2A.Z enrichment were modest, as compared with accessibility changes, and previous Figure S2D appeared to indicate that accessibility slightly increased at sites where H2A.Z decreased. During revision we performed an orthogonal measurement of these data. Rather than quantifying data in aggregate, we quantified accessibly changes at H2A.Z peaks as a distribution. The result of this new analysis can be found in Figure 2D, and data indicate that accessibility in fact did not increase at these regions.
>
> With regard to normalization methods, we could not identify a robust method for normalizing based on background signal, because H2A.Z ChIP-Seq datasets used in our study lacked internal controls. While our RPKM based normalization approach has the potential to cause additional false negatives, as noted by Reviewer 2, it has the advantage of limiting false positives, which was an equally important issue in our analyses.

3. Positional hierarchy of H2A.Z at promoters coincides with differences in gene function.
a. Change in H2A.Z upon ANP32E loss is shown (Figure 3A). It is unclear why genes on forward strand and reverse strand are plotted separately instead of being combined and plotted in the direction of transcription. Secondly, the overall effect of ANP32E loss on H2A.Z is only a ~5-6% increase in the promoter - and it is not clear if this increase is statistically significant. This weak change in H2A.Z upon loss of ANP32E indicates that the dramatic effects on accessibility may be independent of changes in H2A.Z occupancy.

We thank Reviewer 2 for these comments. For the first point, we agree that combining forward and reverse strand plots simplifies the data presentation, so we have now modified figures throughout the manuscript to represent direction of transcription with both strands plotted together.

For the second point, we acknowledge that in previous Figure 3A, where H2A.Z changes were measured across all promoters (even non-H2A.Z marked promoters), the effect was fairly modest, and labeling of our axes was unclear. To accommodate the request of Reviewer 2, we have modified Figure 3A so that the y-axis is log10 fold change and the y-axis in Figure 3B-C is RPKM enrichment values. This new labeling strategy makes it clear in Figure 3B, that RPKM increases more than 40% in aggregate (from 9 to 13 RPKM enrichment) at the -1 nucleosome, for promoters where H2A.Z is already present at the +1 nucleosome. We have also performed new analyses (as suggested by Reviewer 3) and find that H2A.Z increases dramatically at the -1 nucleosome for regions with moderate H2A.Z levels in WT cells (the 60-80th percentile). These results comprise Figure S3A and are perfectly aligned with our proposed H2A.Z hierarchy. We have also performed statistical tests measuring these impacts, found in Figure S3B. Finally, to test whether changes in accessibility occurred independent of changes in H2A.Z occupancy, we repeated our ATAC-Seq studies in *Anp32e*-/- cells that were depleted for H2A.Z. Remarkably, we found that much of the ATAC changes we measured in *Anp32e*-/- cells were rescued, with partial H2A.Z knockdown. These results indicate accessibility is indeed dependent on H2A.Z levels. These outcomes are shown in new Figures 4 and 5D.

b. For Figure 3B, y-axis values are absent, so the extent of changes cannot be assessed, and no statistical analysis is presented to show that the differences are significant. Furthermore, it is not clear how "Normalize Frequency" is calculated. In Figure S2C, D, the range is 0 to 1 for all 3 plots - the exact steps of normalization should be described in the methods.

We have now addressed this concern in our response to Reviewer 2 point 3a.

c. For Figure S3C, it is not clear how many promoters belong to each class in WT H2A.Z. Also, it is not clear if changes in proportions are statistically significant.

We thank the reviewer for identifying these errors. We have made the appropriate corrections, and statistical measures of accessibility changes can be found in Figure 3E and the number of regions in each class is included in the legend.

d. For Figure 3C, it is not clear if the p-values for GO enrichments are corrected for multiple testing.

We agree with Reviewer 2. It is indeed important that GO enrichment p-values are corrected for multiple testing. All p-values are now corrected in this manner and we have adjusted figures and text to reflect this.

It is difficult to assess if the analyses in Figure 3, S3 support the Author's conclusions due to missing information pointed above.

We agree that there were several instances where the presentation of figures made interpretation difficult for Figure 3 and S3. We have now adjusted these figures as

requested by Reviewer 2 (as noted above), and modified our methods section so that these data and analysis methods are more transparent.

4. H2A.Z hierarchy changes correspond with accessibility and nucleosome positioning changes.
a. It is difficult to untangle increase in accessibility of +1 nucleosome versus increased positioning of +1 nucleosome with data as presented and described in Figure 4A (y-axis values missing here also). To check changes in positioning, distribution of width of ATAC peak at +1 nucleosome across genes in WT and ANP32E-/- might be more convincing.

We thank Reviewer 2 for the suggestion. The method we used for assessing nucleosome positioning, termed NucleoATAC has been shown previously to measure nucleosome bound regions similar to data from MNase-Seq (Schep 2015). To better characterize nucleosome positioning, in the revised manuscript we provide further evidence for increased nucleosome positioning at the +1 nucleosome, depicted as a heatmap of NucleoATAC data at promoters marked by H2A.Z or lacking H2A.Z (Figure S6C). In this figure the characteristic enrichment periodicity makes it clear that nucleosomes reside on either side of the TSS at H2A.Z marked promoters, whereas overall accessibility is strongest directly over the TSS (Figure 2A). We also investigated peak width, as Reviewer 2 suggests, but found that peaks reside almost exclusively over the TSS, preventing us from assessing nucleosomes at the +1 position. In other words, the very strong signal from non-nucleosomal fragments at the TSS masked signal from nucleosome sized fragments at the +1 and -1 positions, making peak width measurement uninformative. To overcome this, we separated ATAC-Seq reads based on fragment sizes and find that ANP32E loss leads to an increase in both nucleosome-size (greater than 150bp) and non-nucleosome-size (smaller than 150bp) fragments. Results of this new analysis can be found in Figure 4E.

b. The Authors show a correlation of SMARCA4 ChIP-seq and H2A.Z ChIP-seq in WT and propose "these results are consistent with a nucleosome remodeling mediated mechanism where H2A.Z promotes increased nucleosome positioning flanking the TSS to stimulate chromatin accessibility at the NDR". However, these results could also be consistent with loss of SMARCA4 activity in Anp32E-/- leading to increased positioning. Since only correlation in WT is presented, both possibilities should be discussed in the text.

We thank Reviewer 2 for the insightful comments. To further investigate the impact of ANP32E loss of SMARCA4, during revision we used CUT&Tag to measure SMARCA4 enrichment in WT and *Anp32e*-/- MEFs, and we found that SMARCA4 levels increase at the TSS in cells lacking ANP32E. These new results can be found in Figure 6, and provide strong support for our statement that data is “consistent with a nucleosome remodeling mediated mechanism where H2A.Z promotes increased nucleosome positioning flanking the TSS to stimulate chromatin accessibility at the NDR".

5. TF-footprinting increases adjacent to better positioned H2A.Z nucleosomes.
a. Lack of y-axis values in Figure 5A prevent assessment of the magnitude of increase of NNFs due to ANP32E loss - also it should be determined if the increase in NNF is statistically significant using ATAC replicates. In Figure 5B, the change in NNF is shown only for changes from one class of promoters, it is not clear if other classes have

expected changes in NNF. Even for the plot shown the effect is very weak and no statistics are shown.

We thank Reviewer 2 for noting this, and agree that previous Fig. 5A and 5B, were hard to interpret. We have corrected this issue in the revised manuscript, and new figure panels displaying NNF changes can be found throughout the results section. In Figure 3E we depict NNFs at separate classes of WT H2A.Z marked promoters, and provide the appropriate statistical measurement, as requested. In Figure 3F we depict changes in NNFs measured at promoter where H2A.Z classes change, and again we provide statistical measurements of these changes. In Figure 4E, we demonstrate how NNF levels increase upon ANP32E loss, and then NNF levels decrease at these same locations in cells depleted for H2A.Z – statistical tests are included for these measurements as well. In total, these new analyses accommodate the request of Reviewer 2.

b. For Figure 5C, it is not clear what the scale of differential protection scores is - what does a 0.05 differential protection score mean in terms of inferred TF binding? Without this information, it is difficult to assess the effect size. The effect sizes in 5D are extremely weak, indicating minor increases in inferred TF binding due to loss of ANP32E.

Reviewer 2 notes that the measurements of footprint protection are unclear. Motif protection scoring is commonly used for statistical assessment of TF footprinting in studies such as DNase-Seq (statistical equations can be found in Gusmao et al. *Nat Methods.* 2016), and these scores have recently been adapted for HINT-ATAC studies (Li et al. *Genome Research.* 2019). Protection scores correspond with difference in cleavage events between the footprint and flanking regions. We have added text to the manuscript to better describe these measurements. While these differences in footprint protection scores may seem weak, they are indeed significant, and many thousands of regions are changing, with the majority of changes having positive values. This indicates that there is an overall increase in motifs that are protected in ANP32E deficient cells compared with WT cells.

c. Finally, the title of this section indicates binding adjacent to better positioned H2A.Z nucleosomes. However, no analysis is presented comparing positioning of H2A.Z nucleosomes and TF binding as inferred from ATAC-seq.

We thank Reviewer 2 for making this point. The intention here was to highlight how H2A.Z resides at nucleosomes flanking the TSS (Figure 3), while ATAC signal occurs directly over the TSS (Figure 2). Reviewer 2 is correct in noting that no analysis was done directly comparing nucleosome positioning with TF binding. As such, we agree that the section title was not accurate and it has now been removed. The new section title reflects the addition of new results, as suggested by reviewers and reads "ANP32E loss leads to increased SMARCA4 enrichment and improved nucleosome positioning**."**

6. ANP32E loss leads to dysregulation of genes involved in differentiation.
a. "Consistent with H2A.Z functioning as an activator, gene promoters with increased accessibility tended to be up-regulated, and promoters with reduced accessibility tended to be down-regulated (Figure 6B)." The comparison shown in 6B is between accessibility and gene expression, hence it is not clear why this says anything about H2A.Z as an

activator. Furthermore, this data doesn't rule out the possibility that gene activity sets H2A.Z levels. Hence, both possibilities should be discussed.

We thank Reviewer 2 for making these distinctions. For the first point, we agree with their assessment that we only showed associations between accessibility and gene expression, and we have now made changes in the text to reflect this. For the second point, the reviewer notes that H2A.Z enrichment changes may be a downstream consequence of ANP32E mediated changes rather than an upstream cause of these changes. We have added a brief discussion of this, in the context of our results, within the discussion section.

b. There are robust changes in expression (many genes with Log2FC>1 in Figure 6A), but median log2FC in top quintile of ATAC accessibility is barely above 0, indicating very low correlation of ATAC changes with RNA changes as presented.

Reviewer 2 notes that dramatic increases in accessibility do not associate with major gene expression changes. It is important to note that for many highly correlated biological processes, small changes in one factor (such as chromatin accessibility) often correspond with dramatic changes in another factor (such as gene expression). Thus, we did not expect there to be a direct 1-to-1 relationship between chromatin changes and gene expression changes in our study. Furthermore, differences in magnitude for our measurements could have arisen through biological factors or through methodological differences. In our study, data were acquired using RNA-Seq and ATAC-Seq, and these methods have inherently different degrees of sensitivity and drastically different dynamic ranges. Because RNA-Seq methods are more robust, they have the ability to measure at a much higher sensitivity than ATAC-Seq methods, resulting in a greater magnitude of measurable change. This difference in magnitude could also be the results of many additional biological factors contributing to overall gene expression levels. In our study, we do indeed find that gene expression increases are strongest (with high statistical significance) at regions where chromatin accessibility increases the most (Figure 7B & 7C), indicating that the changes we observed are indeed correlated.

c. In Figure 6D, it is not clear if GO enrichment p-values were corrected for multiple testing. Multiple testing corrections is essential for all GO analyses in the study.

We thank Reviewer 2 for reiterating this important point. All p-values are corrected for multiple testing and we have adjusted figures text to reflect this.

Additional Comments:

1. For sake of reproducibility of the analyses, information on use of published data should be expanded beyond just citations. The exact GEO id for each external dataset used should be part of methods or as a Supplementary Table.

We thank Reviewer 2 for this suggestion, and we have now included this table as a supplemental file.

2. For all line plots and genomic snapshots, y axis needs to be present and methods should describe how the values on the y axes were arrived at.

We have now included additional y-axis details and provide descriptions for how browser snapshots were obtained in the methods section.

Reviewer 3:
Main Concerns:

i. Figure 4
a. “these results are consistent with a nucleosome remodeling mediated mechanism where H2A.Z promotes increased nucleosome positioning flanking the TSS to stimulate chromatin accessibility at the NDR.” i. Shown previously in Figure S2 B-D, accessibility increases regardless of changes in H2A.Z. This figure needs a control analysis to show that loss (or no change) of H2A.Z does not also increase nucleosome positioning at promoters. Conclusions made here and downstream may need be reworked depending on the results of this control analysis.

We thank Reviewer 3 for making note of this issue, and would like to first highlight how our new analyses (new Figure 2D) indicate that chromatin accessibility does not in fact increase at sites where H2A.Z decreases, in spite of the previously displayed aggerate plots suggesting otherwise in Figure S2. The reviewer’s point remains however, and we preformed additional analyses to accommodate this point. Here, we measured nucleosome position changes with regard to H2A.Z at promoters, and found that nucleosome positioning increased at H2A.Z marked promoters, but there was no increase at promoters lacking H2A.Z (Figure S6C & S6D). Additionally, we measured nucleosome positioning at promoters where H2A.Z increased compared with promoters where H2A.Z decreased. Here we found nucleosome positioning increased considerably across promoters where H2A.Z increased, and only modest/variable changes occurred across promoters where H2A.Z decreased (Figure S6E).

ii. Figure 3D
a. “One interesting possibility is that ANP32E levels and H2A.Z positioning are important for defining cellular function during differentiation and potentially for regulating H2A.Z positioning during cellular transitions. Consistent with this possibility, Anp32e mRNA is considerably lower in MEFs compared with MESCs (Figure 3D), and H2A.Z is much more restricted to the +1 nucleosome position in MESCs compared with MEFs”
i. Postulations made using Figure 3D could be further validated by experiments involving overexpression of ANP32E in MEF and observing H2A.Z/accessibility changes at select candidate loci.

We appreciate this suggestion from Reviewer 3. As we began preparing for such experiments during revision, we found it difficult to properly control these *in vitro* over-expression studies, and were unable to address potential artifacts generated from over-expression. In prior studies, ANP32E was functionally defined as a regulator of genomic H2A.Z levels (Murphy et al. 2018, Mao et al. 2014, Obri et al. 2014), therefore, an experiment orthogonal to *Anp32e* over-expression would be to deplete H2A.Z levels. This is precisely what we have done during revision. In Figure 4, we demonstrate that knockdown of H2A.Z causes many of the increases in chromatin accessibility in *Anp32e*-/- cells to be reversed. Understandably, it remains unknown whether ANP32E levels are differentially regulated during mESC differentiation, and thus, we hope to investigate this hypothesis in future follow-up studies.

iii. Figure 3
a. Seems like there was some opportunity lost for some reciprocal validation here in Figure 3.
i. For instance, if you looked at promoters in the top 25% of +1 nucleosome H2A.Z enrichment in WT, you would expect to see H2A.Z spread to the -1 nucleosome at these same promoters in Anp32e-/-

> We thank Reviewer 3 for this excellent suggestion. This was indeed an opportunity lost, and we have now made this correction. In the revised manuscript we have performed the analysis suggested by Reviewer 3, and our results are just as Reviewer 3 predicted. Here, we parsed H2A.Z marked regions based on enrichment levels, and find that H2A.Z does indeed spread to the -1 nucleosome for promoters with moderate H2A.Z levels, and not for promoters with minimal or high H2A.Z levels. This completely orthogonal analysis result aligns perfectly with our proposed H2A.Z hierarchy strategy – suggesting that H2A.Z levels were already at "saturation" for promoters with the highest H2A.Z levels in WT, and H2A.Z spreads to the -1 position for promoters transitioning from Class One to Class Two in *Anp32e*-/- cells. These results can be found in Figure S3A. We would like to reiterate our appreciation for this excellent suggestion.

iv. Figure 5
a. The authors showed that NNFs accumulated more at promoters that transitioned from Class One to Class Two in Anp32e-/-. They then identified several motifs with positive HINT scores, indicating increased TF binding. However, the authors fail to connect these putative changes in TF binding back to changes in H2A.Z enrichment and therefore fail to reinforce their conclusions on H2A.Z promoting nucleosome positioning established in the previous figure.

> We thank Reviewer 3 for these suggestions. As indicated, we did indeed demonstrate that NNFs increased at these promoter regions transitioning from Class One to Class Two, and then we identified putative TF binding increases that occurred in the *Anp32e*-/- cells. While these results provided initial evidence that TF binding changes occurred in a manner dependent on H2A.Z, we sought a more definitive means to connect putative TF binding changes with H2A.Z changes. Thus, we performed a series of new experiments measuring changes in NNF enrichment after H2A.Z depletion in *Anp32e*-/- cells. We found that the previously measured increases in NNFs were mostly reversed, and NNFs enrichment was reduced upon H2A.Z knockdown. This occurred at WT H2A.Z sites, but did not occur at promoters lacking H2A.Z (new Figure 4D & 4E). Additionally, we repeated our HINT-ATAC measurements assessing motif protection in *Anp32e*-/- cells in which H2A.Z had been depleted. Here we found that protection scores for the majority of the statistically significant positive HINT-ATAC motifs became negative upon H2A.Z depletion (new Figure 5D). This further suggests that TF binding is impacted by ANP32E in an H2A.Z dependent manner.

b. Additionally, this figure in particular would benefit from ChIP-qPCR experiments for candidate TFs to validate the proposed increased TF binding inferred by the HINT analysis.

> We thank Reviewer 3 for this suggestion. In the revised version of the manuscript we have used CUT&Tag to validate that SP1 binding increases in the ANP32E lacking

cells. SP1 binding increased at the same genomic SP1 motifs where accessibility increased. These results can be found in Figure 5B, S5A, and S5B.

Minor Concerns

v. Introduction
a. The first paragraph of the intro seems quite overwrought. For example, the statement that “whether particular factors regulate widespread chromatin state remains largely unknown” is simply not true. Likewise, for the sentence, “…removal of factors thought to function in regulation of chromatin accessibility, including H3K27ac, H3.3, and prominent nucleosome remodelers, had only modest impacts on genome-wide chromatin states”. Aside from the issue of defining what a “modest” impact is, the two references cited in this sentence could be counterbalanced by many studies of remodelers, polycomb group proteins, and histone variants like cenH3, that do indeed have widespread effects on chromatin. Perhaps a better use of space here is to simply introduce the general topic in a more even-handed way.

We thank the review for this suggestion and we have now made these changes.

vi. Figure 1E
a. What defines an accessible/H2A.Z enriched promoter? Peak overlap? If so, by how much?

Overlapping accessible/H2A.Z enriched promoters were defined by any degree of peak overlap. This description is now included in the methods section.

vii. Figure S1D
a. “These results indicate that H2A.Z is consistently correlated with chromatin accessibility, as well as with marks of gene activation H3K4me3 and H3K27ac, but H2A.Z is only associated with the gene silencing mark H3K27me3 in the context of bivalent chromatin, at a subset of locations in MESCs.” i. I don’t think this claim can be appropriately made based on how the data are presented.

Upon further consideration, we agree that this claim was not accurate and so we have now changed the text to read “These results indicate that H2A.Z is consistently correlated with chromatin accessibility, as well as with marks of gene activation H3K4me3 and H3K27ac, but H2A.Z is poorly associated with the gene silencing mark H3K27me3 in differentiated MEFs.”

viii. Figure 2B-C
a. Reduced H2AZ and increased accessibility “represented the extreme minority of regions, and chromatin changes at these sites were much less significant than changes at all other sites”. i. First, this is almost a quarter of all peaks. ii. Second, “much less significant” implies statistical analysis. Consider rephrasing.

With new analysis suggested by Review 2, we now find that regions where H2A.Z is lost do not display a statistically significant increase in accessibility (Figure 2D). As a result of these new measurements, the text referenced by Reviewer 3 has been eliminated.

ix. Figure 3A - Include “H2A.Z” in plot title to orient reader. b. Consider changing y-axis to fold change as opposed to percentage.

These suggested changes have been made.

x. Figure S3A
a. Several analyses in the paper are divided into +/- strand transcription. This division should be justified in the text.

In the revised manuscript genes on the +/- strand are now combined and oriented in the direction of transcription from left to right.

xi. Figure 3B
a. Figure 3B depicts C1 to C2 (top) and C0 to C1 (bottom) transitions. However, Figure 3C depicts GO analysis for C1->C2 and C1->C0 transitions. Displayed in this way, the reader has no insight into what a 1->0 transition looks like from WT to Anp32e-/- cells.
b. Here and many other places, the y-axis is labeled ‘normalize frequency”, but this should be “normalized frequency.”

As noted by Reviewer 3, the measurement related to promoter transitions from C1 to C0 seemed unnecessary and these have now been eliminated. We have also more accurately labeled all y-axes throughout the manuscript.

xii. Figure 6B
a. “Consistent with H2A.Z functioning as an activator, gene promoters with increased accessibility tended to be up-regulated, and promoters with reduced accessibility tended to be down-regulated” i. How exactly is that consistent with H2AZ’s function as an activator? Analysis in Figure 2 showed positive or negative changes in H2A.Z lead to increased accessibility.

This comment is similar to comments from Reviewer 2 point 6. We have now corrected the text to better articulate relationships between accessibility and gene expression.

xiii. Figure 6C
a. “Furthermore, we observed a more dramatic gene expression change when we restricted our analysis to genes in closest proximity to intergenic motifs identified by footprinting analysis (Figure 6C), indicating that the ANP32E mediated changes at intergenic regulatory regions, such as enhancers, may be a major contributor to the gene expression changes we observed.” i. Axis should be consistent between B and C to avoid misleading the reader.

After further measurements, we find that gene expression changes correlate equally well with overall accessibility and accessibility at distal footprint sites. We have corrected the text to reflect the correct interpretation of our data.

xiv. Discussion
a. “Class Zero promoters lack H2A.Z, have poorly positioned nucleosomes, and are largely inaccessible.” i. Class Zero does lack H2AZ but nucleosome position was never

analyzed at this class.

We have now assessed nucleosome position and accessibility changes at promoters lacking H2A.Z (Class Zero) – Figure 2A, Figure S6C-E.

b. "Furthermore, H2A.Z marked gene promoters that gained more H2A.Z in the absence of ANP32E, and became more accessibility, tended to be up-regulated"
i. Typo: accessibility -> accessible

We have now corrected this typo and thank Reviewer 3 for altering us to this error.

c. Claims in discussion should cite relevant Figures

We have now incorporated references to figures throughout the discussion.

xv. Method
a. "Promoters with increased H2A.Z at +1 nucleosome are defined as log2FC values in top 25% at -1 nucleosome and bottom 75% at +1 nucleosome."
i. Typo: should say "top 25% at +1 nucleosomes and bottom 75% at -1 nucleosomes."

This was a critical mistake in the methods section and we are very thankful that Reviewer 3 noticed this error. It has now been corrected.

b. "Data were compared with data from studies which utilized Chromatin Immuno-Precipitation Sequencing (ChIP-Seq), but no new ChIP-Seq datasets were generated from this study. Data from numerous published studies were utilized 24,32,64–66."
i. Each dataset used should be more explicitly stated in text.

As suggested by other Reviewers as well, we have now included a supplemental table listing all GEO datasets utilized.

Reviewer #1 (Remarks to the Author):

This is a revised manuscript from Murphy et al regarding the role of ANP32E in positioning H2AZ with downstream consequences for chromatin accessibility and gene regulation. The major addition in revision is the knockdown of H2AZ in ANP32E null MEF and subsequent ATAC-seq. This experiment is performed because the changes in H2AZ enrichment in ANP32E null MEF are relatively modest compared to changes in chromatin accessibility. Since H2AZ KD in ANP32E null MEF "rescues" the chromatin accessibility differences (although the comparison to WT is not made here), the authors attribute the impact to H2AZ. They further provide validation of TF footprinting results with SP1 enrichment profiling in WT and ANP32E null MEF. Generally, these new data seem sound.

There were some concerns that were not really addressed, both I think for my questions as well as concerns of the other reviewers. For example, in the first submission, the authors show that enhancers and promoters seem similarly enriched with Smarca4, but H2AZ enrichment is clearly higher at promoters compared with enhancers. The response here was simply to combine these regions in analysis. But I'm still not sure how this delineated observation fits with the model that H2AZ deposition provides preferential sites of either Smarca4 recruitment or remodeling activity to promote open chromatin. Also, the authors never really address the observation that a good number of sites move from C2->C1 or C1->C0 in ANP32E null MEFs as shown in Fig. S3C. Regardless, I do think there are a number of interesting observations and analyses performed that warrant publication.

Reviewer #2 (Remarks to the Author):

The authors have substantially improved the manuscript with revisions. The H2A.Z knockdown experiments support the main conclusions of the manuscript.

Minor comment:
SP1 footprints are stronger after removing H2A.Z, which moves in the same direction as Anp32e KO compared to WT. This should be pointed out in the Results section especially since this is the factor that was profiled by CUT&TAG in Anp32e KO cells. For at least SP1, it seems like the pathways promoting its binding are more complicated than just increase in H2A.Z upon loss of Anp32e.

Reviewer #3 (Remarks to the Author):

The authors have addressed my previous concerns in their revision. The amount of extra experimental and analytical work they carried out in revision process is really commendable. I am happy to support publication of this work.

All reviewers seem quite satisfied with our revisions to the manuscript, and 2 out of the 3 (Reviewers 1 and 3) indicate that the revised manuscript now warrants publication. Reviewer 2 requests a minor change to the text in the results section, and although Reviewer 1 does indicate their approval for publication, they also note some minor concerns. We have addressed all concerns during this final round of revisions.

Reviewer 1 Comment:

This is a revised manuscript from Murphy et al regarding the role of ANP32E in positioning H2AZ with downstream consequences for chromatin accessibility and gene regulation. The major addition in revision is the knockdown of H2AZ in ANP32E null MEF and subsequent ATAC-seq. This experiment is performed because the changes in H2AZ enrichment in ANP32E null MEF are relatively modest compared to changes in chromatin accessibility. Since H2AZ KD in ANP32E null MEF "rescues" the chromatin accessibility differences (although the comparison to WT is not made here), the authors attribute the impact to H2AZ. They further provide validation of TF footprinting results with SP1 enrichment profiling in WT and ANP32E null MEF. Generally, these new data seem sound.

There were some concerns that were not really addressed, both I think for my questions as well as concerns of the other reviewers. For example, in the first submission, the authors show that enhancers and promoters seem similarly enriched with Smarca4, but H2AZ enrichment is clearly higher at promoters compared with enhancers. The response here was simply to combine these regions in analysis. But I'm still not sure how this delineated observation fits with the model that H2AZ deposition provides preferential sites of either Smarca4 recruitment or remodeling activity to promote open chromatin. Also, the authors never really address the observation that a good number of sites move from C2->C1 or C1->C0 in ANP32E null MEFs as shown in Fig. S3C. Regardless, I do think there are a number of interesting observations and analyses performed that warrant publication.

Responses:

1) Reviewer 1 indicates that the revisions to our manuscript warrant publication, but they do make note of minor changes that could improve our manuscript. They note that we eliminated our analysis of SMARCA4 and H2A.Z at enhancer regions, and they refer to their earlier concern "if the model is that SMARCA4 is more likely to be bound at an H2AZ nucleosome, what about the relatively inverted binding pattern observed for enhancers and promoters for H2AZ and SMARCA4?" To accommodate this request, we now include the enhancer analysis that was present in our initial submission and omitted during our earlier revision. We indicate that SMARCA4 and H2A.Z are indeed correlated at enhancers, but this correlation is not as strong as promoters. These results demonstrate that the binding of H2A.Z and SMARCA4 are not "inverted", as Reviewer 1 suggests. We refer to our new supplemental figure 6C with the following statement: "The correlation between SMARCA4 and H2A.Z was more moderate at enhancers compared with promoters (Fig S6C)."

2) Reviewer 1 indicates that "the authors never really address the observation that a good number of sites move from C2->C1 or C1->C0 in ANP32E null MEFs as shown in Fig. S3C". Here they are referring to a prior concern from Reviewer 3 – who now indicates that the manuscript warrants publication. Reviewer 3 previously stated "the reader has no insight into what a 1->0 transition looks like from WT to Anp32e-/- cells". To accommodate this request, we now provide gene ontology analysis (new supplemental figure 3E) for promoters that change from C2->C1 or C1->C0 in ANP32E null MEFs. This analysis was done during our initial submission, but eliminated during the earlier

revision process. We refer to new supplemental figure 3E in the text with the statement: "expansion of H2A.Z to the -1 nucleosome (going from Class One to Class Two) in *Anp32e* null MEFs occurred at genes associated with developmental differentiation and mRNA translation (Fig S3D), while reduction in H2A.Z was associated with proliferation, locomotion, and migration (Fig S3E)."

Reviewer 2 Comment:

The authors have substantially improved the manuscript with revisions. The H2A.Z knockdown experiments support the main conclusions of the manuscript.

Minor comment: SP1 footprints are stronger after removing H2A.Z, which moves in the same direction as Anp32e KO compared to WT. This should be pointed out in the Results section especially since this is the factor that was profiled by CUT&TAG in Anp32e KO cells. For at least SP1, it seems like the pathways promoting its binding are more complicated than just increase in H2A.Z upon loss of Anp32e.

Response:

To accommodate this concern from Reviewer 2, we have modified the text in the results section to include the following sentence: "Motifs for SP1 were not among this group, suggesting that additional factors impact SP1 binding."

Reviewer 3 Comment:

The authors have addressed my previous concerns in their revision. The amount of extra experimental and analytical work they carried out in revision process is really commendable. I am happy to support publication of this work.
